# Supplementary material for: Prognosis prediction of uterine cervical cancer using changes in the histogram and texture features of apparent diffusion coefficient during definitive chemoradiotherapy
Source: PLoS One. 2023 Mar 31;18(3):e0282710. doi: 10.1371/journal.pone.0282710 (PMC10065283; doi:10.1371/journal.pone.0282710)

**Supporting information**

**S1 Fig. Kaplan-Meier plots for SqCC patients.**

Log-rank *p* values were calculated.

*Abbreviations:* glcm = Grey level co-occurrence matrix;


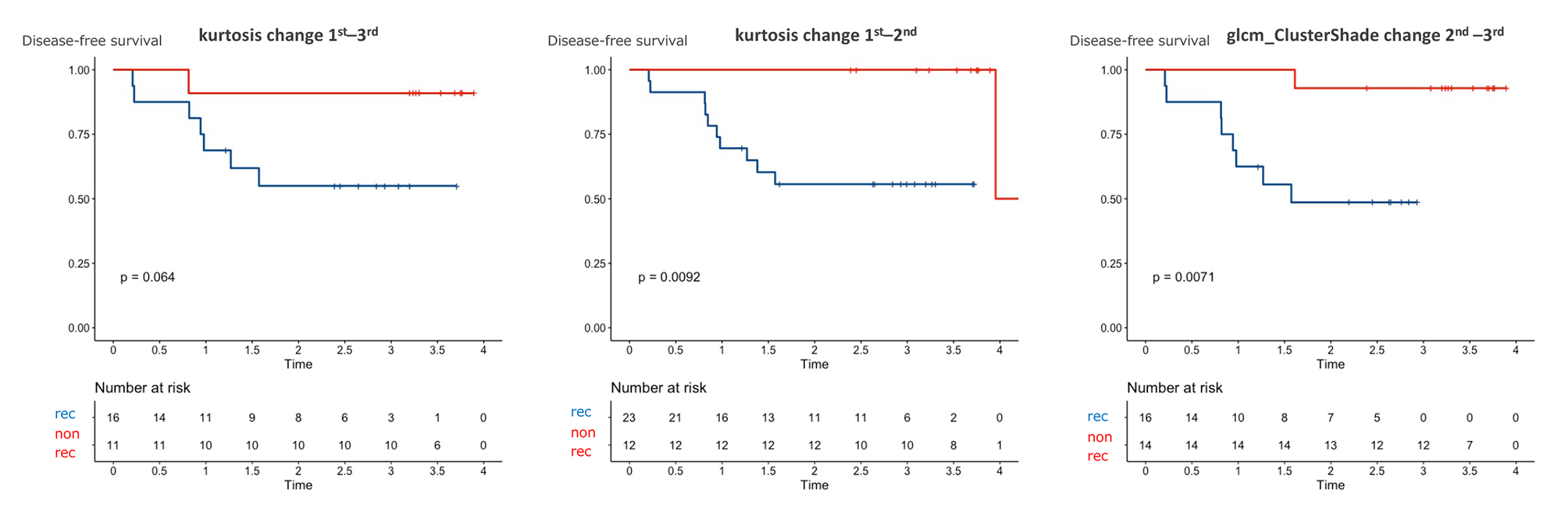

Supplement: S1 Fig — Log-rank p values were calculated. Abbreviations: glcm = grey level co-occurrence matrix. (DOCX) [file pone.0282710.s001.docx]
