# Supplementary material for: Prognosis prediction of uterine cervical cancer using changes in the histogram and texture features of apparent diffusion coefficient during definitive chemoradiotherapy
Source: PLoS One. 2023 Mar 31;18(3):e0282710. doi: 10.1371/journal.pone.0282710 (PMC10065283; doi:10.1371/journal.pone.0282710)
Supplement: S1 Table — (DOCX) [file pone.0282710.s002.docx]

**Supporting information**

**S1 Table.** **Clinical data and the results of univariate analyses of SqCC patients only.**

|  |  | All = 49 | Rec = 17 | Non-rec = 32 | p |
| --- | --- | --- | --- | --- | --- |
| Site of recurrence | local |  | 5 |  |  |
|  | distant |  | 12 |  |  |
| Age (mean ± SD) |  | 61.7 ± 14.1 | 61.9 ± 12.6 | 61.6 ± 14.9 | 1 |
| FIGO | 1B | 12 | 3 | 9 | 0.755 |
|  | 2A | 8 | 4 | 4 |  |
|  | 2B | 13 | 4 | 9 |  |
|  | 3A | 2 | 1 | 1 |  |
|  | 3B | 11 | 4 | 7 |  |
|  | 4A | 3 | 1 | 2 |  |

*Abbreviations: rec* = recurrent, non-rec= non-recurrent

Forty-nine of 57 patiens had SqCC. Of these, 17 suffered recurrences (S1 Table).

The results of the ROC analysis in the 49 patients with SqCC at 2y are shown in S2 Table. The glcm_ClusterShade change 2^nd^–3^rd^ subgroups showed the highest area under the curve (AUC = 0.815). The kurtosis change 1^st^–3^rd^ and 1^st^–2^nd^ subgroups also showed high AUCs (0.775 and 0.763, respectively). We determined the cut-off values for these three change rates and created Kaplan-Meier plots (Fig.S1).
