# Supplementary material for: Prognosis prediction of uterine cervical cancer using changes in the histogram and texture features of apparent diffusion coefficient during definitive chemoradiotherapy
Source: PLoS One. 2023 Mar 31;18(3):e0282710. doi: 10.1371/journal.pone.0282710 (PMC10065283; doi:10.1371/journal.pone.0282710)
Supplement: S2 Table — (DOCX) [file pone.0282710.s003.docx]

**Supporting information**

**S2 Table. The image parameters with high AUCs in the 49 SqCC patients**.

| **image parameters** | **status** | **AUC** |
| --- | --- | --- |
| glcm_ClusterShade | change rate 2^nd^–3^rd^ | 0.815 |
| shape_Elongation | change rate 3^rd^–4^th^ | 0.793 |
| shape_Elongation | change rate 1^st^–3^rd^ | 0.775 |
| Kurtosis | change rate 1^st^–3^rd^ | 0.775 |
| Kurtosis | change rate 1^st^–2^nd^ | 0.763 |
| Skewness | change rate 2^nd^–3^rd^ | 0.756 |
| ngtdm_Strength | 4^th^ | 0.753 |
| shape_Flatness | 1^st^ | 0.752 |
| glcm_Id | 1^st^ | 0.741 |
| glcm_Idm | 1^st^ | 0.741 |

*Abbreviations:* AUC = area under the curve glcm = Grey level co-occurrence matrix;; ngtdm = neighbourhood grey tone difference matrix; glcm_Id= Grey level co-occurrence matrix Inverse Difference; glcm_Idm= Grey level co-occurrence matrix Inverse Difference Moment
