# Supplementary material for: Prognosis prediction of uterine cervical cancer using changes in the histogram and texture features of apparent diffusion coefficient during definitive chemoradiotherapy
Source: PLoS One. 2023 Mar 31;18(3):e0282710. doi: 10.1371/journal.pone.0282710 (PMC10065283; doi:10.1371/journal.pone.0282710)
Supplement: S4 Table — (DOCX) [file pone.0282710.s005.docx]

**Supporting information**

**S4 Table. The AUCs of all image parameters.**

| image features_all patients | AUC |
| --- | --- |
| original_firstorder_Kurtosis.3.1 | 0.785 |
| original_firstorder_Kurtosis.1st–2nd | 0.759 |
| original_glcm_ClusterShade.2nd–3rd | 0.750 |
| original_glcm_Id.1 | 0.737 |
| original_glcm_Imc2.1st–3rd | 0.736 |
| original_glcm_Idm.1 | 0.734 |
| original_gldm_DependenceVariance.1 | 0.734 |
| original_shape_SurfaceVolumeRatio.2 | 0.733 |
| original_gldm_DependenceNonUniformityNormalized.1 | 0.732 |
| original_glszm_LargeAreaHighGrayLevelEmphasis.3.1 | 0.729 |
| original_ngtdm_Coarseness.3.1 | 0.729 |
| original_glcm_SumSquares.1st–2nd | 0.727 |
| original_glcm_InverseVariance.1 | 0.727 |
| original_glcm_Contrast.1 | 0.725 |
| original_gldm_LargeDependenceEmphasis.1 | 0.725 |
| original_glszm_GrayLevelVariance.1 | 0.725 |
| original_glcm_DifferenceAverage.1 | 0.722 |
| original_glrlm_RunVariance.1 | 0.722 |
| original_shape_SurfaceArea.3.1 | 0.722 |
| original_glrlm_RunLengthNonUniformity.3.1 | 0.722 |
| original_ngtdm_Strength.4 | 0.721 |
| original_glrlm_LongRunEmphasis.1 | 0.720 |
| original_gldm_GrayLevelVariance.1 | 0.717 |
| original_shape_MeshVolume.3.1 | 0.715 |
| original_shape_MinorAxisLength.3.1 | 0.715 |
| original_shape_VoxelVolume.3.1 | 0.715 |
| original_firstorder_Energy.3.1 | 0.715 |
| original_glcm_DifferenceVariance.1 | 0.715 |
| original_glrlm_RunLengthNonUniformityNormalized.1 | 0.715 |
| original_glrlm_RunPercentage.1 | 0.715 |
| original_glrlm_ShortRunEmphasis.1 | 0.715 |
| original_glcm_ClusterTendency.1st–2nd | 0.714 |
| original_firstorder_Variance.1 | 0.713 |
| original_shape_Maximum2DDiameterSlice.2nd–3rd | 0.713 |
| original_ngtdm_Contrast.3rd–4th | 0.713 |
| original_glrlm_GrayLevelVariance.1 | 0.710 |
| original_glszm_ZonePercentage.4 | 0.710 |
| original_glcm_DifferenceAverage.1st–2nd | 0.709 |
| original_shape_Elongation.3.1 | 0.708 |
| original_firstorder_TotalEnergy.3.1 | 0.708 |
| original_glrlm_RunLengthNonUniformityNormalized.3.1 | 0.708 |
| original_glrlm_RunPercentage.3.1 | 0.708 |
| original_glrlm_ShortRunEmphasis.3.1 | 0.708 |
| original_glrlm_GrayLevelNonUniformity.3.1 | 0.708 |
| original_glszm_GrayLevelNonUniformity.3rd–4th | 0.706 |
| original_firstorder_Uniformity.1st–2nd | 0.705 |
| original_glcm_Idn.1st–2nd | 0.705 |
| original_glszm_GrayLevelNonUniformityNormalized.1st–2nd | 0.705 |
| original_glcm_SumSquares.1 | 0.700 |
| original_glcm_InverseVariance.1st–2nd | 0.700 |
| original_ngtdm_Coarseness.4 | 0.699 |
| original_glszm_GrayLevelNonUniformityNormalized.1 | 0.698 |
| original_glcm_ClusterProminence.1 | 0.696 |
| original_firstorder_MeanAbsoluteDeviation.1st–2nd | 0.695 |
| original_glcm_ClusterProminence.1st–2nd | 0.695 |
| original_glcm_Contrast.1st–2nd | 0.695 |
| original_glrlm_GrayLevelNonUniformityNormalized.1st–2nd | 0.695 |
| original_gldm_DependenceEntropy.2 | 0.695 |
| original_ngtdm_Busyness.2nd–4th | 0.695 |
| original_gldm_DependenceNonUniformity.3.1 | 0.694 |
| original_gldm_GrayLevelNonUniformity.3.1 | 0.694 |
| original_glszm_LargeAreaEmphasis.3.1 | 0.694 |
| original_firstorder_10Percentile.4.1 | 0.694 |
| original_firstorder_InterquartileRange.1st–2nd | 0.691 |
| original_firstorder_RobustMeanAbsoluteDeviation.1st–2nd | 0.691 |
| original_glcm_ClusterShade.1st–2nd | 0.691 |
| original_glszm_ZoneEntropy.1st–2nd | 0.691 |
| original_firstorder_MeanAbsoluteDeviation.1 | 0.691 |
| original_gldm_SmallDependenceHighGrayLevelEmphasis.1 | 0.691 |
| original_glszm_LargeAreaLowGrayLevelEmphasis.1 | 0.691 |
| original_gldm_DependenceVariance.2 | 0.690 |
| original_glrlm_RunLengthNonUniformityNormalized.2 | 0.690 |
| original_glszm_LargeAreaEmphasis.2 | 0.690 |
| original_gldm_LargeDependenceLowGrayLevelEmphasis.4 | 0.688 |
| original_glcm_Correlation.2 | 0.688 |
| original_shape_LeastAxisLength.3.1 | 0.688 |
| original_glszm_GrayLevelNonUniformity.3.1 | 0.688 |
| original_glcm_DifferenceEntropy.1 | 0.684 |
| original_glrlm_RunPercentage.2 | 0.683 |
| original_glrlm_ShortRunEmphasis.2 | 0.683 |
| original_firstorder_Skewness.2nd–3rd | 0.681 |
| original_shape_Flatness.1 | 0.681 |
| original_gldm_LargeDependenceLowGrayLevelEmphasis.1 | 0.681 |
| original_gldm_SmallDependenceEmphasis.1 | 0.681 |
| original_glcm_Idn.3.1 | 0.681 |
| original_glszm_ZoneVariance.3.1 | 0.681 |
| original_firstorder_90Percentile.4 | 0.680 |
| original_shape_LeastAxisLength.2 | 0.680 |
| original_glszm_LargeAreaLowGrayLevelEmphasis.2 | 0.680 |
| original_gldm_LargeDependenceEmphasis.2 | 0.680 |
| original_gldm_SmallDependenceEmphasis.2 | 0.680 |
| original_glcm_ClusterTendency.1 | 0.679 |
| original_ngtdm_Contrast.1 | 0.679 |
| original_shape_MeshVolume.2 | 0.678 |
| original_firstorder_Entropy.1st–2nd | 0.677 |
| original_firstorder_Variance.1st–2nd | 0.677 |
| original_gldm_GrayLevelVariance.1st–2nd | 0.677 |
| original_glrlm_GrayLevelVariance.1st–2nd | 0.677 |
| original_glrlm_RunEntropy.1st–2nd | 0.677 |
| original_glrlm_GrayLevelNonUniformityNormalized.1 | 0.676 |
| original_glcm_ClusterProminence.2nd–3rd | 0.675 |
| original_gldm_GrayLevelVariance.2nd–3rd | 0.675 |
| original_glrlm_GrayLevelVariance.2nd–3rd | 0.675 |
| original_shape_Elongation.3rd–4th | 0.675 |
| original_glcm_ClusterShade.3rd–4th | 0.675 |
| original_firstorder_TotalEnergy.2 | 0.675 |
| original_gldm_DependenceNonUniformityNormalized.2 | 0.675 |
| original_glrlm_LongRunEmphasis.4 | 0.674 |
| original_firstorder_Entropy.1 | 0.674 |
| original_gldm_DependenceVariance.3.1 | 0.674 |
| original_glrlm_RunVariance.4 | 0.673 |
| original_firstorder_Maximum.1st–2nd | 0.673 |
| original_glcm_DifferenceVariance.1st–2nd | 0.673 |
| original_glcm_Id.1st–2nd | 0.673 |
| original_ngtdm_Contrast.1st–2nd | 0.673 |
| original_gldm_DependenceNonUniformity.2nd–4th | 0.673 |
| original_shape_VoxelVolume.2 | 0.670 |
| original_glrlm_RunPercentage.4 | 0.669 |
| original_firstorder_Uniformity.1 | 0.669 |
| original_firstorder_Variance.2nd–3rd | 0.669 |
| original_glszm_GrayLevelVariance.2nd–3rd | 0.669 |
| original_glcm_Correlation.1st–2nd | 0.668 |
| original_glcm_Idm.1st–2nd | 0.668 |
| original_firstorder_Maximum.4 | 0.667 |
| original_shape_Elongation.1 | 0.667 |
| original_ngtdm_Coarseness.1 | 0.667 |
| original_firstorder_Maximum.3.1 | 0.667 |
| original_glrlm_LongRunEmphasis.3.1 | 0.667 |
| original_glszm_LargeAreaLowGrayLevelEmphasis.4 | 0.666 |
| original_firstorder_RobustMeanAbsoluteDeviation.1 | 0.664 |
| original_glszm_ZonePercentage.1 | 0.664 |
| original_glszm_GrayLevelVariance.1st–2nd | 0.664 |
| original_glcm_MaximumProbability.1st–2nd | 0.664 |
| original_glcm_SumEntropy.1st–2nd | 0.664 |
| original_glrlm_LongRunEmphasis.2 | 0.663 |
| original_gldm_DependenceNonUniformity.2nd–3rd | 0.663 |
| original_glszm_GrayLevelNonUniformityNormalized.2nd–3rd | 0.663 |
| original_glcm_ClusterTendency.3rd–4th | 0.663 |
| original_glcm_InverseVariance.3rd–4th | 0.663 |
| original_glcm_Imc2.1 | 0.662 |
| original_shape_Maximum3DDiameter.2nd–4th | 0.660 |
| original_shape_SurfaceVolumeRatio.2nd–4th | 0.660 |
| original_glrlm_ShortRunEmphasis.4 | 0.660 |
| original_glszm_SizeZoneNonUniformity.3.1 | 0.660 |
| original_glcm_SumEntropy.1 | 0.659 |
| original_glcm_DifferenceEntropy.1st–2nd | 0.659 |
| original_glcm_Idmn.1st–2nd | 0.659 |
| original_glrlm_RunLengthNonUniformityNormalized.4 | 0.658 |
| original_glcm_Imc2.2 | 0.658 |
| original_glrlm_RunVariance.2 | 0.658 |
| original_ngtdm_Strength.1 | 0.657 |
| original_glrlm_RunEntropy.2nd–3rd | 0.656 |
| original_firstorder_MeanAbsoluteDeviation.3rd–4th | 0.656 |
| original_glrlm_GrayLevelNonUniformity.3rd–4th | 0.656 |
| original_ngtdm_Strength.3rd–4th | 0.656 |
| original_firstorder_Maximum.4.1 | 0.656 |
| original_firstorder_Mean.4 | 0.655 |
| original_firstorder_RootMeanSquared.4 | 0.655 |
| original_firstorder_InterquartileRange.1 | 0.655 |
| original_glcm_JointEnergy.1st–2nd | 0.655 |
| original_glcm_JointEntropy.1st–2nd | 0.655 |
| original_ngtdm_Strength.3 | 0.653 |
| original_glszm_LargeAreaHighGrayLevelEmphasis.4.1 | 0.653 |
| original_glcm_Imc1.1st–3rd | 0.653 |
| original_gldm_DependenceNonUniformityNormalized.3.1 | 0.653 |
| original_glszm_SizeZoneNonUniformityNormalized.3.1 | 0.653 |
| original_glszm_SmallAreaEmphasis.3.1 | 0.653 |
| original_firstorder_Energy.2 | 0.653 |
| original_glcm_Imc1.2 | 0.653 |
| original_shape_Maximum2DDiameterColumn.2nd–4th | 0.653 |
| original_shape_Maximum2DDiameterSlice.2nd–4th | 0.653 |
| original_gldm_GrayLevelNonUniformity.2 | 0.650 |
| original_glcm_Imc2.1st–2nd | 0.650 |
| original_glszm_ZoneVariance.1st–2nd | 0.650 |
| original_shape_Elongation.2nd–3rd | 0.650 |
| original_glszm_SizeZoneNonUniformity.2nd–4th | 0.650 |
| original_glszm_SmallAreaEmphasis.2nd–4th | 0.650 |
| original_glrlm_GrayLevelVariance.3rd–4th | 0.650 |
| original_glrlm_RunEntropy.3rd–4th | 0.650 |
| original_firstorder_Kurtosis.4 | 0.648 |
| original_gldm_SmallDependenceHighGrayLevelEmphasis.4 | 0.648 |
| original_glszm_LargeAreaHighGrayLevelEmphasis.2 | 0.648 |
| original_glszm_ZoneVariance.2 | 0.648 |
| original_glszm_ZoneVariance.1 | 0.647 |
| original_firstorder_Median.4 | 0.647 |
| original_shape_Sphericity.3.1 | 0.646 |
| original_shape_SurfaceVolumeRatio.3.1 | 0.646 |
| original_gldm_LargeDependenceHighGrayLevelEmphasis.3.1 | 0.646 |
| original_firstorder_Range.3.1 | 0.646 |
| original_gldm_LargeDependenceEmphasis.3.1 | 0.646 |
| original_shape_SurfaceArea.2 | 0.645 |
| original_glrlm_GrayLevelNonUniformity.2 | 0.645 |
| original_glszm_SizeZoneNonUniformityNormalized.2 | 0.645 |
| original_glcm_Idmn.1 | 0.645 |
| original_glcm_MaximumProbability.1 | 0.645 |
| original_gldm_GrayLevelNonUniformity.1 | 0.645 |
| original_firstorder_MeanAbsoluteDeviation.2nd–3rd | 0.644 |
| original_firstorder_Range.2nd–3rd | 0.644 |
| original_glcm_ClusterTendency.2nd–3rd | 0.644 |
| original_glszm_SizeZoneNonUniformity.2nd–3rd | 0.644 |
| original_firstorder_Variance.3rd–4th | 0.644 |
| original_glcm_ClusterProminence.3rd–4th | 0.644 |
| original_firstorder_10Percentile.4 | 0.644 |
| original_firstorder_Energy.2nd–4th | 0.643 |
| original_firstorder_TotalEnergy.2nd–4th | 0.643 |
| original_shape_MajorAxisLength.2nd–4th | 0.643 |
| original_shape_MeshVolume.2nd–4th | 0.643 |
| original_shape_VoxelVolume.2nd–4th | 0.643 |
| original_glszm_SmallAreaEmphasis.3 | 0.641 |
| original_firstorder_Range.3 | 0.641 |
| original_gldm_SmallDependenceEmphasis.3 | 0.641 |
| original_ngtdm_Coarseness.1st–2nd | 0.641 |
| original_ngtdm_Contrast.4 | 0.641 |
| original_glrlm_GrayLevelNonUniformity.1 | 0.640 |
| original_glszm_LargeAreaEmphasis.1 | 0.640 |
| original_ngtdm_Busyness.1 | 0.640 |
| original_shape_Maximum2DDiameterSlice.2 | 0.640 |
| original_glcm_JointAverage.4 | 0.639 |
| original_glcm_SumAverage.4 | 0.639 |
| original_firstorder_Kurtosis.4.1 | 0.638 |
| original_gldm_DependenceVariance.4 | 0.638 |
| original_gldm_SmallDependenceLowGrayLevelEmphasis.2 | 0.638 |
| original_glcm_Imc2.2nd–3rd | 0.638 |
| original_glcm_SumSquares.2nd–3rd | 0.638 |
| original_glrlm_RunLengthNonUniformity.2nd–4th | 0.638 |
| original_gldm_GrayLevelVariance.3rd–4th | 0.638 |
| original_glszm_GrayLevelVariance.3rd–4th | 0.638 |
| original_glcm_Autocorrelation.4 | 0.636 |
| original_glszm_LargeAreaHighGrayLevelEmphasis.1st–2nd | 0.636 |
| original_glcm_Imc2.3 | 0.635 |
| original_glszm_SizeZoneNonUniformityNormalized.3 | 0.635 |
| original_glszm_ZonePercentage.3 | 0.635 |
| original_shape_LeastAxisLength.1 | 0.635 |
| original_glcm_Idn.1 | 0.635 |
| original_glszm_SmallAreaLowGrayLevelEmphasis.4 | 0.635 |
| original_glcm_ClusterShade.1 | 0.633 |
| original_glszm_LargeAreaHighGrayLevelEmphasis.1 | 0.633 |
| original_glszm_SmallAreaEmphasis.2 | 0.633 |
| original_glrlm_RunLengthNonUniformity.2 | 0.633 |
| original_shape_SurfaceVolumeRatio.4 | 0.632 |
| original_glszm_ZonePercentage.3.1 | 0.632 |
| original_gldm_GrayLevelNonUniformity.1st–2nd | 0.632 |
| original_glrlm_GrayLevelNonUniformity.1st–2nd | 0.632 |
| original_gldm_GrayLevelNonUniformity.3rd–4th | 0.631 |
| original_glrlm_ShortRunHighGrayLevelEmphasis.1 | 0.630 |
| original_glrlm_GrayLevelNonUniformity.2nd–4th | 0.630 |
| original_glrlm_RunEntropy.2nd–4th | 0.630 |
| original_glcm_Correlation.3 | 0.629 |
| original_glcm_DifferenceVariance.3 | 0.629 |
| original_glcm_MCC.3 | 0.629 |
| original_firstorder_Median.4.1 | 0.629 |
| original_glcm_DifferenceVariance.4 | 0.629 |
| original_shape_SurfaceVolumeRatio.1 | 0.628 |
| original_gldm_GrayLevelNonUniformity.2nd–4th | 0.628 |
| original_glszm_LargeAreaEmphasis.1st–2nd | 0.627 |
| original_firstorder_TotalEnergy.4.1 | 0.626 |
| original_glcm_JointEnergy.1 | 0.626 |
| original_glcm_JointEntropy.1 | 0.626 |
| original_gldm_DependenceNonUniformity.2 | 0.625 |
| original_shape_Flatness.3.1 | 0.625 |
| original_glcm_Correlation.3.1 | 0.625 |
| original_gldm_SmallDependenceLowGrayLevelEmphasis.3.1 | 0.625 |
| original_glszm_LargeAreaLowGrayLevelEmphasis.3.1 | 0.625 |
| original_ngtdm_Busyness.3.1 | 0.625 |
| original_shape_MinorAxisLength.2nd–3rd | 0.625 |
| original_glszm_LargeAreaHighGrayLevelEmphasis.2nd–3rd | 0.625 |
| original_glszm_SmallAreaHighGrayLevelEmphasis.2nd–3rd | 0.625 |
| original_ngtdm_Contrast.2nd–3rd | 0.625 |
| original_firstorder_Range.2nd–4th | 0.625 |
| original_glszm_LargeAreaHighGrayLevelEmphasis.2nd–4th | 0.625 |
| original_glszm_SizeZoneNonUniformityNormalized.2nd–4th | 0.625 |
| original_firstorder_RobustMeanAbsoluteDeviation.3rd–4th | 0.625 |
| original_glrlm_ShortRunHighGrayLevelEmphasis.4 | 0.625 |
| original_glszm_SmallAreaEmphasis.4 | 0.625 |
| original_shape_SurfaceVolumeRatio.4.1 | 0.624 |
| original_firstorder_Mean.4.1 | 0.624 |
| original_gldm_SmallDependenceEmphasis.4 | 0.623 |
| original_glrlm_HighGrayLevelRunEmphasis.4 | 0.623 |
| original_glcm_MCC.2 | 0.623 |
| original_glszm_LargeAreaEmphasis.2nd–4th | 0.623 |
| original_gldm_HighGrayLevelEmphasis.4 | 0.622 |
| original_gldm_LargeDependenceEmphasis.4 | 0.622 |
| original_gldm_HighGrayLevelEmphasis.1 | 0.621 |
| original_glszm_LargeAreaEmphasis.4.1 | 0.621 |
| original_glszm_HighGrayLevelZoneEmphasis.4 | 0.620 |
| original_glszm_GrayLevelNonUniformity.2 | 0.620 |
| original_ngtdm_Busyness.2 | 0.620 |
| original_shape_SurfaceArea.2nd–4th | 0.620 |
| original_glszm_SizeZoneNonUniformityNormalized.4 | 0.619 |
| original_glcm_Contrast.2nd–3rd | 0.619 |
| original_glcm_DifferenceAverage.2nd–3rd | 0.619 |
| original_glrlm_ShortRunEmphasis.2nd–3rd | 0.619 |
| original_glrlm_HighGrayLevelRunEmphasis.1 | 0.618 |
| original_glrlm_RunPercentage.1st–2nd | 0.618 |
| original_shape_Maximum2DDiameterSlice.3.1 | 0.618 |
| original_glcm_Idmn.3.1 | 0.618 |
| original_gldm_SmallDependenceHighGrayLevelEmphasis.3 | 0.618 |
| original_shape_LeastAxisLength.4.1 | 0.618 |
| original_shape_Maximum2DDiameterColumn.4.1 | 0.618 |
| original_shape_Maximum3DDiameter.2 | 0.618 |
| original_glszm_GrayLevelNonUniformityNormalized.2nd–4th | 0.618 |
| original_shape_MeshVolume.1 | 0.616 |
| original_glszm_ZonePercentage.2 | 0.615 |
| original_firstorder_Energy.4.1 | 0.615 |
| original_shape_Flatness.1st–2nd | 0.614 |
| original_glrlm_ShortRunEmphasis.1st–2nd | 0.614 |
| original_glszm_GrayLevelNonUniformity.1st–2nd | 0.614 |
| original_shape_VoxelVolume.1 | 0.614 |
| original_glcm_Autocorrelation.1 | 0.614 |
| original_glcm_MCC.4 | 0.613 |
| original_shape_MinorAxisLength.2 | 0.613 |
| original_shape_Sphericity.2nd–3rd | 0.613 |
| original_shape_SurfaceArea.2nd–3rd | 0.613 |
| original_glcm_InverseVariance.2nd–3rd | 0.613 |
| original_glszm_ZoneEntropy.2nd–3rd | 0.613 |
| original_shape_Maximum3DDiameter.3rd–4th | 0.613 |
| original_shape_MeshVolume.3rd–4th | 0.613 |
| original_glcm_Imc2.3rd–4th | 0.613 |
| original_gldm_DependenceNonUniformity.3rd–4th | 0.613 |
| original_glszm_LargeAreaHighGrayLevelEmphasis.3rd–4th | 0.613 |
| original_shape_VoxelVolume.2nd–3rd | 0.613 |
| original_firstorder_Minimum.3 | 0.612 |
| original_ngtdm_Busyness.3 | 0.612 |
| original_gldm_GrayLevelVariance.3 | 0.612 |
| original_glszm_GrayLevelVariance.3 | 0.612 |
| original_firstorder_Skewness.1 | 0.611 |
| original_glrlm_RunEntropy.1 | 0.611 |
| original_glszm_GrayLevelNonUniformity.1 | 0.611 |
| original_glcm_ClusterShade.3.1 | 0.611 |
| original_shape_Flatness.2 | 0.610 |
| original_glszm_SizeZoneNonUniformity.2 | 0.610 |
| original_glszm_GrayLevelVariance.2nd–4th | 0.610 |
| original_glszm_HighGrayLevelZoneEmphasis.2nd–4th | 0.610 |
| original_glrlm_LongRunEmphasis.1st–2nd | 0.609 |
| original_glrlm_RunLengthNonUniformityNormalized.1st–2nd | 0.609 |
| original_glcm_Imc2.1st–4th | 0.609 |
| original_gldm_GrayLevelNonUniformity.4.1 | 0.609 |
| original_glrlm_GrayLevelNonUniformity.4.1 | 0.609 |
| original_firstorder_TotalEnergy.1 | 0.609 |
| original_glcm_JointAverage.1 | 0.609 |
| original_glcm_SumAverage.1 | 0.609 |
| original_glszm_LargeAreaEmphasis.4 | 0.609 |
| original_shape_Maximum2DDiameterColumn.2 | 0.608 |
| original_glcm_Idm.2 | 0.608 |
| original_ngtdm_Coarseness.2 | 0.608 |
| original_shape_Flatness.4 | 0.607 |
| original_glszm_ZoneVariance.2nd–3rd | 0.606 |
| original_firstorder_Maximum.2nd–3rd | 0.606 |
| original_firstorder_Minimum.2nd–3rd | 0.606 |
| original_firstorder_RobustMeanAbsoluteDeviation.2nd–3rd | 0.606 |
| original_glcm_DifferenceVariance.2nd–3rd | 0.606 |
| original_glrlm_GrayLevelNonUniformityNormalized.2nd–3rd | 0.606 |
| original_glrlm_RunLengthNonUniformityNormalized.2nd–3rd | 0.606 |
| original_glrlm_RunLengthNonUniformity.3rd–4th | 0.606 |
| original_firstorder_Variance.3 | 0.606 |
| original_glcm_ClusterProminence.3 | 0.606 |
| original_glcm_Contrast.3 | 0.606 |
| original_glrlm_GrayLevelVariance.3 | 0.606 |
| original_glrlm_RunVariance.3 | 0.606 |
| original_firstorder_RootMeanSquared.4.1 | 0.606 |
| original_firstorder_Skewness.4.1 | 0.606 |
| original_shape_MeshVolume.4 | 0.606 |
| original_glcm_Id.2 | 0.605 |
| original_gldm_LargeDependenceHighGrayLevelEmphasis.2 | 0.605 |
| original_glcm_MCC.3.1 | 0.604 |
| original_glrlm_RunVariance.3.1 | 0.604 |
| original_glrlm_LongRunHighGrayLevelEmphasis.4 | 0.604 |
| original_shape_SurfaceArea.1 | 0.604 |
| original_firstorder_Energy.1 | 0.604 |
| original_glrlm_RunLengthNonUniformity.1 | 0.604 |
| original_shape_MeshVolume.4.1 | 0.603 |
| original_firstorder_Minimum.1 | 0.603 |
| original_glcm_Imc2.2nd–4th | 0.603 |
| original_glszm_GrayLevelNonUniformity.2nd–4th | 0.603 |
| original_glszm_ZoneVariance.2nd–4th | 0.603 |
| original_glszm_HighGrayLevelZoneEmphasis.1 | 0.601 |
| original_glcm_Contrast.4 | 0.601 |
| original_firstorder_MeanAbsoluteDeviation.3 | 0.600 |
| original_glcm_Imc1.3 | 0.600 |
| original_glrlm_HighGrayLevelRunEmphasis.3 | 0.600 |
| original_glrlm_ShortRunHighGrayLevelEmphasis.3 | 0.600 |
| original_glszm_HighGrayLevelZoneEmphasis.3 | 0.600 |
| original_glszm_ZoneVariance.3 | 0.600 |
| original_shape_VoxelVolume.4.1 | 0.600 |
| original_glcm_ClusterShade.4.1 | 0.600 |
| original_glrlm_RunPercentage.4.1 | 0.600 |
| original_glrlm_ShortRunEmphasis.4.1 | 0.600 |
| original_glszm_ZoneVariance.4.1 | 0.600 |
| original_gldm_DependenceVariance.2nd–3rd | 0.600 |
| original_glrlm_RunLengthNonUniformity.2nd–3rd | 0.600 |
| original_glcm_Imc1.2nd–4th | 0.600 |
| original_glcm_JointAverage.2nd–4th | 0.600 |
| original_glcm_SumAverage.2nd–4th | 0.600 |
| original_ngtdm_Coarseness.2nd–4th | 0.600 |
| original_shape_SurfaceVolumeRatio.3rd–4th | 0.600 |
| original_shape_VoxelVolume.3rd–4th | 0.600 |
| original_firstorder_TotalEnergy.3rd–4th | 0.600 |
| original_glcm_SumSquares.3rd–4th | 0.600 |
| original_glrlm_RunLengthNonUniformityNormalized.3rd–4th | 0.600 |
| original_firstorder_Entropy.4 | 0.600 |
| original_glrlm_LongRunLowGrayLevelEmphasis.4 | 0.600 |
| original_shape_MinorAxisLength.1 | 0.599 |
| original_firstorder_Uniformity.4 | 0.598 |
| original_glcm_InverseVariance.2 | 0.598 |
| original_glcm_Autocorrelation.2nd–4th | 0.598 |
| original_gldm_SmallDependenceEmphasis.3.1 | 0.597 |
| original_glrlm_RunLengthNonUniformityNormalized.4.1 | 0.597 |
| original_shape_VoxelVolume.4 | 0.597 |
| original_glcm_Imc1.1 | 0.597 |
| original_shape_LeastAxisLength.1st–2nd | 0.595 |
| original_glszm_ZonePercentage.1st–2nd | 0.595 |
| original_firstorder_Range.4 | 0.595 |
| original_glrlm_GrayLevelNonUniformityNormalized.4 | 0.595 |
| original_glszm_GrayLevelVariance.4 | 0.595 |
| original_gldm_LargeDependenceLowGrayLevelEmphasis.2 | 0.595 |
| original_glcm_InverseVariance.2nd–4th | 0.595 |
| original_glcm_InverseVariance.3 | 0.594 |
| original_gldm_HighGrayLevelEmphasis.3 | 0.594 |
| original_glrlm_LongRunHighGrayLevelEmphasis.3 | 0.594 |
| original_glszm_GrayLevelNonUniformity.4.1 | 0.594 |
| original_glcm_SumSquares.4 | 0.594 |
| original_gldm_DependenceNonUniformityNormalized.4 | 0.594 |
| original_firstorder_Minimum.3.1 | 0.594 |
| original_firstorder_Entropy.2nd–3rd | 0.594 |
| original_firstorder_InterquartileRange.2nd–3rd | 0.594 |
| original_glcm_Correlation.2nd–3rd | 0.594 |
| original_gldm_SmallDependenceHighGrayLevelEmphasis.2nd–3rd | 0.594 |
| original_glszm_LargeAreaEmphasis.2nd–3rd | 0.594 |
| original_ngtdm_Complexity.2nd–3rd | 0.594 |
| original_firstorder_Energy.3rd–4th | 0.594 |
| original_firstorder_InterquartileRange.3rd–4th | 0.594 |
| original_glszm_SmallAreaHighGrayLevelEmphasis.3rd–4th | 0.594 |
| original_firstorder_90Percentile.1 | 0.593 |
| original_shape_Maximum2DDiameterRow.2 | 0.593 |
| original_glcm_ClusterShade.2 | 0.593 |
| original_glrlm_RunEntropy.2 | 0.593 |
| original_ngtdm_Strength.2 | 0.593 |
| original_glrlm_RunLengthNonUniformityNormalized.2nd–4th | 0.593 |
| original_firstorder_Variance.4 | 0.592 |
| original_gldm_DependenceEntropy.1 | 0.592 |
| original_shape_SurfaceArea.4.1 | 0.591 |
| original_gldm_DependenceNonUniformityNormalized.4.1 | 0.591 |
| original_gldm_LargeDependenceEmphasis.4.1 | 0.591 |
| original_glrlm_RunLengthNonUniformity.4.1 | 0.591 |
| original_glszm_SizeZoneNonUniformityNormalized.1st–2nd | 0.591 |
| original_shape_Maximum2DDiameterColumn.3.1 | 0.590 |
| original_firstorder_Uniformity.3.1 | 0.590 |
| original_glcm_ClusterTendency.3.1 | 0.590 |
| original_shape_Sphericity.2 | 0.590 |
| original_glcm_Idn.2 | 0.590 |
| original_firstorder_Maximum.2nd–4th | 0.590 |
| original_gldm_DependenceEntropy.2nd–4th | 0.590 |
| original_gldm_SmallDependenceLowGrayLevelEmphasis.2nd–4th | 0.590 |
| original_glrlm_HighGrayLevelRunEmphasis.2nd–4th | 0.590 |
| original_glrlm_RunPercentage.2nd–4th | 0.590 |
| original_glrlm_ShortRunEmphasis.2nd–4th | 0.590 |
| original_glrlm_ShortRunHighGrayLevelEmphasis.2nd–4th | 0.590 |
| original_glcm_Correlation.4 | 0.589 |
| original_shape_Maximum2DDiameterColumn.1 | 0.589 |
| original_glcm_DifferenceAverage.3 | 0.588 |
| original_glrlm_GrayLevelNonUniformity.3 | 0.588 |
| original_glszm_LargeAreaEmphasis.3 | 0.588 |
| original_glszm_SmallAreaHighGrayLevelEmphasis.3 | 0.588 |
| original_ngtdm_Contrast.3 | 0.588 |
| original_shape_LeastAxisLength.4 | 0.588 |
| original_glcm_Imc2.4 | 0.588 |
| original_glcm_Idmn.2 | 0.588 |
| original_firstorder_10Percentile.2nd–3rd | 0.588 |
| original_firstorder_Uniformity.2nd–3rd | 0.588 |
| original_glcm_Imc1.2nd–3rd | 0.588 |
| original_glrlm_LongRunHighGrayLevelEmphasis.2nd–4th | 0.588 |
| original_glszm_LowGrayLevelZoneEmphasis.2nd–4th | 0.588 |
| original_ngtdm_Contrast.2nd–4th | 0.588 |
| original_glcm_DifferenceEntropy.3rd–4th | 0.588 |
| original_glrlm_RunPercentage.3rd–4th | 0.588 |
| original_glrlm_ShortRunEmphasis.3rd–4th | 0.588 |
| original_glszm_SizeZoneNonUniformity.3rd–4th | 0.588 |
| original_ngtdm_Complexity.3rd–4th | 0.588 |
| original_glcm_ClusterProminence.4 | 0.587 |
| original_glrlm_GrayLevelVariance.4 | 0.587 |
| original_shape_Elongation.1st–2nd | 0.586 |
| original_firstorder_Range.1st–2nd | 0.586 |
| original_gldm_DependenceEntropy.1st–2nd | 0.586 |
| original_gldm_DependenceNonUniformityNormalized.1st–2nd | 0.586 |
| original_gldm_LargeDependenceHighGrayLevelEmphasis.1st–2nd | 0.586 |
| original_glrlm_RunVariance.1st–2nd | 0.586 |
| original_shape_Maximum2DDiameterColumn.3 | 0.585 |
| original_shape_Maximum2DDiameterRow.3 | 0.585 |
| original_glcm_ClusterTendency.4.1 | 0.585 |
| original_glrlm_LongRunEmphasis.4.1 | 0.585 |
| original_gldm_GrayLevelVariance.4 | 0.585 |
| original_glcm_DifferenceAverage.2 | 0.585 |
| original_glcm_Contrast.2 | 0.585 |
| original_glcm_ClusterShade.2nd–4th | 0.585 |
| original_gldm_HighGrayLevelEmphasis.2nd–4th | 0.585 |
| original_glszm_LargeAreaLowGrayLevelEmphasis.2nd–4th | 0.585 |
| original_gldm_DependenceNonUniformity.1 | 0.585 |
| original_glrlm_LongRunHighGrayLevelEmphasis.1 | 0.585 |
| original_glcm_Imc1.4 | 0.584 |
| original_glcm_MaximumProbability.3.1 | 0.583 |
| original_glszm_ZoneEntropy.2 | 0.583 |
| original_shape_LeastAxisLength.2nd–4th | 0.583 |
| original_firstorder_10Percentile.2nd–4th | 0.583 |
| original_glcm_DifferenceVariance.2nd–4th | 0.583 |
| original_gldm_GrayLevelNonUniformity.3 | 0.582 |
| original_shape_Flatness.4.1 | 0.582 |
| original_gldm_SmallDependenceEmphasis.4.1 | 0.582 |
| original_glszm_SizeZoneNonUniformity.4.1 | 0.582 |
| original_glcm_SumSquares.3 | 0.582 |
| original_gldm_LargeDependenceLowGrayLevelEmphasis.3 | 0.582 |
| original_glrlm_LongRunEmphasis.3 | 0.582 |
| original_glszm_LargeAreaLowGrayLevelEmphasis.3 | 0.582 |
| original_firstorder_Kurtosis.1 | 0.582 |
| original_glszm_SmallAreaEmphasis.1st–2nd | 0.582 |
| original_shape_MeshVolume.2nd–3rd | 0.581 |
| original_firstorder_Energy.2nd–3rd | 0.581 |
| original_firstorder_TotalEnergy.2nd–3rd | 0.581 |
| original_glszm_HighGrayLevelZoneEmphasis.2nd–3rd | 0.581 |
| original_ngtdm_Coarseness.2nd–3rd | 0.581 |
| original_firstorder_Entropy.3rd–4th | 0.581 |
| original_gldm_DependenceVariance.3rd–4th | 0.581 |
| original_gldm_LargeDependenceHighGrayLevelEmphasis.3rd–4th | 0.581 |
| original_glrlm_GrayLevelNonUniformityNormalized.3rd–4th | 0.581 |
| original_glszm_GrayLevelNonUniformityNormalized.3rd–4th | 0.581 |
| original_shape_MajorAxisLength.2 | 0.580 |
| original_glcm_SumEntropy.2 | 0.580 |
| original_glcm_ClusterProminence.2nd–4th | 0.580 |
| original_gldm_LowGrayLevelEmphasis.2nd–4th | 0.580 |
| original_glrlm_LowGrayLevelRunEmphasis.2nd–4th | 0.580 |
| original_glrlm_ShortRunLowGrayLevelEmphasis.2nd–4th | 0.580 |
| original_glszm_GrayLevelNonUniformityNormalized.4.1 | 0.579 |
| original_ngtdm_Coarseness.4.1 | 0.579 |
| original_glcm_JointEntropy.4.1 | 0.579 |
| original_glszm_SmallAreaLowGrayLevelEmphasis.2 | 0.578 |
| original_shape_MinorAxisLength.1st–2nd | 0.577 |
| original_gldm_SmallDependenceLowGrayLevelEmphasis.1st–2nd | 0.577 |
| original_glrlm_ShortRunLowGrayLevelEmphasis.1st–2nd | 0.577 |
| original_glcm_ClusterShade.3 | 0.576 |
| original_shape_Maximum2DDiameterSlice.4.1 | 0.576 |
| original_gldm_DependenceVariance.4.1 | 0.576 |
| original_shape_SurfaceVolumeRatio.3 | 0.576 |
| original_glcm_Id.3 | 0.576 |
| original_glcm_Idm.3 | 0.576 |
| original_gldm_LargeDependenceEmphasis.3 | 0.576 |
| original_ngtdm_Complexity.3 | 0.576 |
| original_glcm_ClusterProminence.4.1 | 0.576 |
| original_glszm_LargeAreaLowGrayLevelEmphasis.4.1 | 0.576 |
| original_firstorder_90Percentile.3.1 | 0.576 |
| original_gldm_LargeDependenceLowGrayLevelEmphasis.3.1 | 0.576 |
| original_glrlm_GrayLevelNonUniformityNormalized.3.1 | 0.576 |
| original_ngtdm_Contrast.3.1 | 0.576 |
| original_shape_Maximum2DDiameterColumn.2nd–3rd | 0.575 |
| original_gldm_SmallDependenceHighGrayLevelEmphasis.2nd–4th | 0.575 |
| original_glcm_Contrast.3rd–4th | 0.575 |
| original_shape_Flatness.2nd–3rd | 0.575 |
| original_glrlm_RunPercentage.2nd–3rd | 0.575 |
| original_shape_SurfaceArea.3rd–4th | 0.575 |
| original_firstorder_Uniformity.3rd–4th | 0.575 |
| original_glcm_DifferenceVariance.3rd–4th | 0.575 |
| original_shape_Maximum2DDiameterSlice.1 | 0.574 |
| original_gldm_DependenceNonUniformity.4.1 | 0.574 |
| original_shape_Maximum2DDiameterRow.1st–2nd | 0.573 |
| original_shape_SurfaceVolumeRatio.1st–2nd | 0.573 |
| original_firstorder_10Percentile.1st–2nd | 0.573 |
| original_gldm_SmallDependenceEmphasis.1st–2nd | 0.573 |
| original_glrlm_LongRunLowGrayLevelEmphasis.1st–2nd | 0.573 |
| original_ngtdm_Busyness.1st–2nd | 0.573 |
| original_glszm_ZoneEntropy.2nd–4th | 0.573 |
| original_gldm_LowGrayLevelEmphasis.4 | 0.572 |
| original_glrlm_LowGrayLevelRunEmphasis.4 | 0.572 |
| original_glszm_LowGrayLevelZoneEmphasis.4 | 0.572 |
| original_firstorder_RobustMeanAbsoluteDeviation.3 | 0.571 |
| original_glcm_JointEnergy.3 | 0.571 |
| original_gldm_DependenceNonUniformityNormalized.3 | 0.571 |
| original_glrlm_RunPercentage.3 | 0.571 |
| original_glrlm_ShortRunEmphasis.3 | 0.571 |
| original_glcm_Idn.4.1 | 0.571 |
| original_glcm_DifferenceAverage.4 | 0.570 |
| original_gldm_LargeDependenceHighGrayLevelEmphasis.1 | 0.570 |
| original_ngtdm_Contrast.2 | 0.570 |
| original_shape_Elongation.2nd–4th | 0.570 |
| original_shape_Maximum2DDiameterRow.2nd–4th | 0.570 |
| original_firstorder_Kurtosis.2nd–4th | 0.570 |
| original_glcm_Idn.2nd–4th | 0.570 |
| original_glcm_Id.3.1 | 0.569 |
| original_glszm_HighGrayLevelZoneEmphasis.3.1 | 0.569 |
| original_glszm_SmallAreaHighGrayLevelEmphasis.3.1 | 0.569 |
| original_shape_LeastAxisLength.2nd–3rd | 0.569 |
| original_gldm_GrayLevelNonUniformity.2nd–3rd | 0.569 |
| original_glszm_HighGrayLevelZoneEmphasis.3rd–4th | 0.569 |
| original_glszm_LargeAreaEmphasis.3rd–4th | 0.569 |
| original_gldm_LargeDependenceEmphasis.1st–2nd | 0.568 |
| original_glrlm_LowGrayLevelRunEmphasis.1st–2nd | 0.568 |
| original_glszm_LowGrayLevelZoneEmphasis.1st–2nd | 0.568 |
| original_ngtdm_Strength.1st–2nd | 0.568 |
| original_glcm_JointEnergy.4.1 | 0.568 |
| original_glcm_SumSquares.4.1 | 0.568 |
| original_glszm_SizeZoneNonUniformityNormalized.4.1 | 0.568 |
| original_glszm_SmallAreaEmphasis.4.1 | 0.568 |
| original_firstorder_90Percentile.2 | 0.568 |
| original_glcm_JointEntropy.2 | 0.568 |
| original_ngtdm_Complexity.2 | 0.568 |
| original_glcm_Idm.2nd–4th | 0.568 |
| original_glrlm_LongRunLowGrayLevelEmphasis.2nd–4th | 0.568 |
| original_glcm_MaximumProbability.4 | 0.567 |
| original_glcm_JointEnergy.4 | 0.566 |
| original_glszm_SizeZoneNonUniformity.1 | 0.565 |
| original_glszm_GrayLevelNonUniformityNormalized.2 | 0.565 |
| original_ngtdm_Strength.2nd–4th | 0.565 |
| original_shape_MajorAxisLength.3 | 0.565 |
| original_shape_MeshVolume.3 | 0.565 |
| original_glrlm_RunLengthNonUniformityNormalized.3 | 0.565 |
| original_glszm_GrayLevelNonUniformity.3 | 0.565 |
| original_firstorder_InterquartileRange.4.1 | 0.565 |
| original_glcm_DifferenceVariance.4.1 | 0.565 |
| original_glrlm_ShortRunLowGrayLevelEmphasis.4 | 0.565 |
| original_shape_VoxelVolume.1st–2nd | 0.564 |
| original_glcm_MCC.1st–2nd | 0.564 |
| original_gldm_LowGrayLevelEmphasis.1st–2nd | 0.564 |
| original_glszm_ZoneEntropy.4 | 0.563 |
| original_glszm_GrayLevelNonUniformityNormalized.4 | 0.563 |
| original_gldm_SmallDependenceLowGrayLevelEmphasis.1 | 0.563 |
| original_firstorder_MeanAbsoluteDeviation.3.1 | 0.563 |
| original_firstorder_RobustMeanAbsoluteDeviation.3.1 | 0.563 |
| original_glcm_Idm.3.1 | 0.563 |
| original_glcm_SumSquares.3.1 | 0.563 |
| original_ngtdm_Strength.3.1 | 0.563 |
| original_shape_MajorAxisLength.2nd–3rd | 0.563 |
| original_shape_SurfaceVolumeRatio.2nd–3rd | 0.563 |
| original_firstorder_Median.2nd–3rd | 0.563 |
| original_glcm_Idn.2nd–3rd | 0.563 |
| original_glrlm_HighGrayLevelRunEmphasis.2nd–3rd | 0.563 |
| original_glszm_GrayLevelNonUniformity.2nd–3rd | 0.563 |
| original_glszm_SizeZoneNonUniformityNormalized.2nd–3rd | 0.563 |
| original_firstorder_Skewness.2nd–4th | 0.563 |
| original_glcm_ClusterTendency.2nd–4th | 0.563 |
| original_glcm_Id.2nd–4th | 0.563 |
| original_glszm_SmallAreaLowGrayLevelEmphasis.2nd–4th | 0.563 |
| original_shape_LeastAxisLength.3rd–4th | 0.563 |
| original_shape_MajorAxisLength.3rd–4th | 0.563 |
| original_glcm_Idmn.3rd–4th | 0.563 |
| original_glcm_Imc1.3rd–4th | 0.563 |
| original_glcm_MaximumProbability.3rd–4th | 0.563 |
| original_ngtdm_Busyness.3rd–4th | 0.563 |
| original_firstorder_RobustMeanAbsoluteDeviation.4.1 | 0.562 |
| original_glcm_SumEntropy.4.1 | 0.562 |
| original_firstorder_Maximum.1 | 0.562 |
| original_glcm_JointEntropy.4 | 0.562 |
| original_firstorder_MeanAbsoluteDeviation.4 | 0.560 |
| original_glrlm_GrayLevelNonUniformityNormalized.2nd–4th | 0.560 |
| original_shape_Maximum3DDiameter.1st–2nd | 0.559 |
| original_shape_MeshVolume.1st–2nd | 0.559 |
| original_shape_Sphericity.1st–2nd | 0.559 |
| original_shape_SurfaceArea.1st–2nd | 0.559 |
| original_glcm_Imc1.1st–2nd | 0.559 |
| original_glszm_SizeZoneNonUniformity.1st–2nd | 0.559 |
| original_ngtdm_Complexity.1st–2nd | 0.559 |
| original_shape_SurfaceArea.3 | 0.559 |
| original_glcm_JointEntropy.3 | 0.559 |
| original_gldm_DependenceVariance.3 | 0.559 |
| original_shape_Maximum3DDiameter.4.1 | 0.559 |
| original_firstorder_90Percentile.4.1 | 0.559 |
| original_glszm_ZonePercentage.4.1 | 0.559 |
| original_glcm_ClusterProminence.2 | 0.558 |
| original_gldm_LargeDependenceLowGrayLevelEmphasis.2nd–4th | 0.558 |
| original_shape_Maximum2DDiameterRow.4 | 0.557 |
| original_firstorder_Mean.2nd–3rd | 0.556 |
| original_glcm_DifferenceEntropy.2nd–3rd | 0.556 |
| original_glcm_Id.2nd–3rd | 0.556 |
| original_gldm_DependenceEntropy.2nd–3rd | 0.556 |
| original_gldm_SmallDependenceLowGrayLevelEmphasis.2nd–3rd | 0.556 |
| original_glrlm_GrayLevelNonUniformity.2nd–3rd | 0.556 |
| original_glrlm_LongRunHighGrayLevelEmphasis.2nd–3rd | 0.556 |
| original_glszm_SmallAreaLowGrayLevelEmphasis.2nd–3rd | 0.556 |
| original_shape_Flatness.3rd–4th | 0.556 |
| original_firstorder_Range.3rd–4th | 0.556 |
| original_gldm_HighGrayLevelEmphasis.3rd–4th | 0.556 |
| original_gldm_LargeDependenceEmphasis.3rd–4th | 0.556 |
| original_gldm_SmallDependenceHighGrayLevelEmphasis.3rd–4th | 0.556 |
| original_glrlm_HighGrayLevelRunEmphasis.3rd–4th | 0.556 |
| original_glrlm_LongRunHighGrayLevelEmphasis.3rd–4th | 0.556 |
| original_glrlm_RunVariance.3rd–4th | 0.556 |
| original_glrlm_ShortRunHighGrayLevelEmphasis.3rd–4th | 0.556 |
| original_ngtdm_Coarseness.3rd–4th | 0.556 |
| original_glrlm_LongRunLowGrayLevelEmphasis.1 | 0.556 |
| original_firstorder_Entropy.3.1 | 0.556 |
| original_firstorder_InterquartileRange.3.1 | 0.556 |
| original_glcm_DifferenceAverage.3.1 | 0.556 |
| original_glcm_JointEnergy.3.1 | 0.556 |
| original_glszm_SmallAreaLowGrayLevelEmphasis.3.1 | 0.556 |
| original_shape_Flatness.2nd–4th | 0.555 |
| original_firstorder_Entropy.2nd–4th | 0.555 |
| original_firstorder_Uniformity.2nd–4th | 0.555 |
| original_gldm_LargeDependenceHighGrayLevelEmphasis.2nd–4th | 0.555 |
| original_glrlm_LongRunEmphasis.2nd–4th | 0.555 |
| original_glszm_ZonePercentage.2nd–4th | 0.555 |
| original_glrlm_RunLengthNonUniformity.1st–2nd | 0.555 |
| original_shape_Elongation.4 | 0.554 |
| original_glcm_ClusterShade.4 | 0.554 |
| original_firstorder_RootMeanSquared.1 | 0.553 |
| original_shape_MinorAxisLength.3 | 0.553 |
| original_firstorder_InterquartileRange.3 | 0.553 |
| original_glcm_ClusterTendency.3 | 0.553 |
| original_glcm_DifferenceEntropy.3 | 0.553 |
| original_glcm_MaximumProbability.3 | 0.553 |
| original_glrlm_RunLengthNonUniformity.3 | 0.553 |
| original_glszm_GrayLevelNonUniformityNormalized.3 | 0.553 |
| original_shape_MinorAxisLength.4.1 | 0.553 |
| original_glszm_SmallAreaHighGrayLevelEmphasis.4.1 | 0.553 |
| original_glcm_Id.4 | 0.553 |
| original_glcm_ClusterTendency.2 | 0.553 |
| original_glcm_JointEnergy.2 | 0.553 |
| original_firstorder_Median.2 | 0.550 |
| original_shape_Maximum3DDiameter.2nd–3rd | 0.550 |
| original_glcm_Idmn.2nd–3rd | 0.550 |
| original_gldm_HighGrayLevelEmphasis.2nd–3rd | 0.550 |
| original_gldm_LargeDependenceEmphasis.2nd–3rd | 0.550 |
| original_gldm_LargeDependenceLowGrayLevelEmphasis.2nd–3rd | 0.550 |
| original_gldm_LowGrayLevelEmphasis.2nd–3rd | 0.550 |
| original_glrlm_LongRunLowGrayLevelEmphasis.2nd–3rd | 0.550 |
| original_glrlm_ShortRunHighGrayLevelEmphasis.2nd–3rd | 0.550 |
| original_firstorder_90Percentile.2nd–4th | 0.550 |
| original_firstorder_Mean.2nd–4th | 0.550 |
| original_glrlm_GrayLevelVariance.2nd–4th | 0.550 |
| original_firstorder_Median.3rd–4th | 0.550 |
| original_glszm_ZonePercentage.3rd–4th | 0.550 |
| original_firstorder_Variance.3.1 | 0.549 |
| original_gldm_GrayLevelVariance.3.1 | 0.549 |
| original_glrlm_LongRunLowGrayLevelEmphasis.3.1 | 0.549 |
| original_firstorder_Minimum.2 | 0.548 |
| original_gldm_DependenceVariance.2nd–4th | 0.548 |
| original_shape_Sphericity.3 | 0.547 |
| original_firstorder_Entropy.3 | 0.547 |
| original_glszm_LargeAreaHighGrayLevelEmphasis.3 | 0.547 |
| original_glszm_SmallAreaLowGrayLevelEmphasis.3 | 0.547 |
| original_ngtdm_Coarseness.3 | 0.547 |
| original_firstorder_Entropy.4.1 | 0.547 |
| original_glcm_Correlation.4.1 | 0.547 |
| original_shape_LeastAxisLength.3 | 0.547 |
| original_shape_VoxelVolume.3 | 0.547 |
| original_firstorder_Maximum.3 | 0.547 |
| original_gldm_SmallDependenceLowGrayLevelEmphasis.3 | 0.547 |
| original_glcm_MaximumProbability.4.1 | 0.547 |
| original_glcm_Idm.4 | 0.547 |
| original_gldm_DependenceVariance.1st–2nd | 0.545 |
| original_firstorder_Kurtosis.2 | 0.545 |
| original_firstorder_RootMeanSquared.2 | 0.545 |
| original_glcm_MaximumProbability.2 | 0.545 |
| original_firstorder_Minimum.2nd–4th | 0.545 |
| original_firstorder_RootMeanSquared.2nd–4th | 0.545 |
| original_gldm_LargeDependenceEmphasis.2nd–4th | 0.545 |
| original_glrlm_GrayLevelNonUniformityNormalized.4.1 | 0.544 |
| original_ngtdm_Complexity.4.1 | 0.544 |
| original_shape_SurfaceArea.4 | 0.544 |
| original_ngtdm_Strength.2nd–3rd | 0.544 |
| original_glcm_JointEntropy.3rd–4th | 0.544 |
| original_glcm_Idm.2nd–3rd | 0.544 |
| original_glcm_MaximumProbability.2nd–3rd | 0.544 |
| original_gldm_LargeDependenceHighGrayLevelEmphasis.2nd–3rd | 0.544 |
| original_glrlm_ShortRunLowGrayLevelEmphasis.2nd–3rd | 0.544 |
| original_shape_Maximum2DDiameterColumn.3rd–4th | 0.544 |
| original_glcm_Idn.3rd–4th | 0.544 |
| original_glrlm_LongRunEmphasis.3rd–4th | 0.544 |
| original_glszm_SmallAreaHighGrayLevelEmphasis.1 | 0.543 |
| original_gldm_GrayLevelVariance.2nd–4th | 0.543 |
| original_shape_Maximum2DDiameterRow.3.1 | 0.542 |
| original_firstorder_10Percentile.3.1 | 0.542 |
| original_glcm_Contrast.3.1 | 0.542 |
| original_glcm_SumEntropy.3.1 | 0.542 |
| original_glrlm_RunEntropy.3 | 0.541 |
| original_shape_Flatness.3 | 0.541 |
| original_firstorder_Mean.3 | 0.541 |
| original_glcm_Autocorrelation.3 | 0.541 |
| original_gldm_DependenceEntropy.3 | 0.541 |
| original_gldm_LowGrayLevelEmphasis.3 | 0.541 |
| original_glrlm_LongRunLowGrayLevelEmphasis.3 | 0.541 |
| original_firstorder_Median.1st–2nd | 0.541 |
| original_glszm_GrayLevelVariance.2 | 0.540 |
| original_firstorder_Median.2nd–4th | 0.540 |
| original_glcm_SumEntropy.4 | 0.540 |
| original_ngtdm_Busyness.4 | 0.540 |
| original_firstorder_Mean.1 | 0.539 |
| original_gldm_LargeDependenceHighGrayLevelEmphasis.4.1 | 0.538 |
| original_shape_MinorAxisLength.4 | 0.538 |
| original_firstorder_Maximum.2 | 0.538 |
| original_firstorder_Skewness.2 | 0.538 |
| original_glszm_HighGrayLevelZoneEmphasis.2 | 0.538 |
| original_glszm_SmallAreaHighGrayLevelEmphasis.2 | 0.538 |
| original_glrlm_LowGrayLevelRunEmphasis.2nd–3rd | 0.538 |
| original_glszm_LowGrayLevelZoneEmphasis.2nd–3rd | 0.538 |
| original_firstorder_Variance.2nd–4th | 0.538 |
| original_glcm_JointEnergy.2nd–4th | 0.538 |
| original_glcm_DifferenceAverage.3rd–4th | 0.538 |
| original_glszm_ZoneVariance.3rd–4th | 0.538 |
| original_glcm_ClusterTendency.4 | 0.537 |
| original_shape_Maximum2DDiameterColumn.1st–2nd | 0.536 |
| original_firstorder_Energy.1st–2nd | 0.536 |
| original_glszm_SmallAreaLowGrayLevelEmphasis.1 | 0.536 |
| original_shape_Maximum2DDiameterSlice.3 | 0.535 |
| original_glcm_JointAverage.3 | 0.535 |
| original_glcm_SumAverage.3 | 0.535 |
| original_glrlm_GrayLevelNonUniformityNormalized.3 | 0.535 |
| original_glrlm_LowGrayLevelRunEmphasis.3 | 0.535 |
| original_glrlm_ShortRunLowGrayLevelEmphasis.3 | 0.535 |
| original_firstorder_Uniformity.4.1 | 0.535 |
| original_firstorder_Entropy.2 | 0.535 |
| original_firstorder_Mean.2 | 0.535 |
| original_firstorder_Range.2 | 0.535 |
| original_glszm_LowGrayLevelZoneEmphasis.2 | 0.535 |
| original_gldm_LowGrayLevelEmphasis.3.1 | 0.535 |
| original_glrlm_LowGrayLevelRunEmphasis.3.1 | 0.535 |
| original_shape_Maximum3DDiameter.1 | 0.534 |
| original_firstorder_RobustMeanAbsoluteDeviation.4 | 0.534 |
| original_firstorder_Median.1 | 0.533 |
| original_firstorder_Energy.4 | 0.532 |
| original_gldm_GrayLevelNonUniformity.4 | 0.532 |
| original_firstorder_Skewness.1st–2nd | 0.532 |
| original_firstorder_TotalEnergy.1st–2nd | 0.532 |
| original_firstorder_RootMeanSquared.2nd–3rd | 0.531 |
| original_glrlm_GrayLevelNonUniformity.4 | 0.531 |
| original_glszm_ZoneVariance.4 | 0.531 |
| original_glrlm_GrayLevelNonUniformityNormalized.2 | 0.530 |
| original_gldm_DependenceNonUniformityNormalized.2nd–4th | 0.530 |
| original_firstorder_90Percentile.3 | 0.529 |
| original_firstorder_Median.3 | 0.529 |
| original_firstorder_RootMeanSquared.3 | 0.529 |
| original_glszm_GrayLevelVariance.4.1 | 0.529 |
| original_ngtdm_Strength.4.1 | 0.529 |
| original_glcm_Idmn.4 | 0.529 |
| original_glrlm_RunEntropy.4 | 0.529 |
| original_firstorder_Minimum.4 | 0.529 |
| original_shape_MajorAxisLength.3.1 | 0.528 |
| original_glcm_JointEntropy.3.1 | 0.528 |
| original_glrlm_GrayLevelVariance.3.1 | 0.528 |
| original_firstorder_Uniformity.2 | 0.528 |
| original_glcm_JointEntropy.2nd–4th | 0.528 |
| original_glcm_JointAverage.1st–2nd | 0.527 |
| original_glcm_SumAverage.1st–2nd | 0.527 |
| original_gldm_LargeDependenceLowGrayLevelEmphasis.1st–2nd | 0.527 |
| original_glszm_LowGrayLevelZoneEmphasis.4.1 | 0.526 |
| original_shape_Maximum2DDiameterRow.2nd–3rd | 0.525 |
| original_gldm_DependenceNonUniformityNormalized.2nd–3rd | 0.525 |
| original_glrlm_LongRunEmphasis.2nd–3rd | 0.525 |
| original_glszm_LargeAreaLowGrayLevelEmphasis.2nd–3rd | 0.525 |
| original_glcm_Id.3rd–4th | 0.525 |
| original_glszm_SizeZoneNonUniformityNormalized.3rd–4th | 0.525 |
| original_shape_MajorAxisLength.1 | 0.524 |
| original_glcm_Correlation.1 | 0.524 |
| original_firstorder_Kurtosis.3 | 0.524 |
| original_firstorder_Uniformity.3 | 0.524 |
| original_gldm_DependenceEntropy.4.1 | 0.524 |
| original_gldm_HighGrayLevelEmphasis.4.1 | 0.524 |
| original_glrlm_RunEntropy.4.1 | 0.524 |
| original_glrlm_ShortRunHighGrayLevelEmphasis.4.1 | 0.524 |
| original_firstorder_Skewness.4 | 0.523 |
| original_gldm_DependenceNonUniformity.1st–2nd | 0.523 |
| original_ngtdm_Complexity.2nd–4th | 0.523 |
| original_shape_Maximum2DDiameterRow.1 | 0.522 |
| original_firstorder_Median.3.1 | 0.521 |
| original_firstorder_RootMeanSquared.3.1 | 0.521 |
| original_glrlm_ShortRunLowGrayLevelEmphasis.3.1 | 0.521 |
| original_glrlm_RunVariance.4.1 | 0.521 |
| original_glszm_GrayLevelNonUniformity.4 | 0.521 |
| original_firstorder_InterquartileRange.2 | 0.520 |
| original_glcm_SumSquares.2 | 0.520 |
| original_shape_MinorAxisLength.2nd–4th | 0.520 |
| original_firstorder_MeanAbsoluteDeviation.2nd–4th | 0.520 |
| original_glszm_ZonePercentage.2nd–3rd | 0.519 |
| original_glcm_Idm.3rd–4th | 0.519 |
| original_glcm_MCC.3rd–4th | 0.519 |
| original_gldm_SmallDependenceEmphasis.3rd–4th | 0.519 |
| original_gldm_SmallDependenceHighGrayLevelEmphasis.1st–2nd | 0.518 |
| original_glrlm_HighGrayLevelRunEmphasis.4.1 | 0.518 |
| original_glszm_ZoneEntropy.4.1 | 0.518 |
| original_glcm_SumEntropy.3 | 0.518 |
| original_glcm_Contrast.2nd–4th | 0.518 |
| original_firstorder_InterquartileRange.2nd–4th | 0.515 |
| original_gldm_SmallDependenceEmphasis.2nd–4th | 0.515 |
| original_glszm_SmallAreaLowGrayLevelEmphasis.4.1 | 0.515 |
| original_glcm_DifferenceEntropy.4.1 | 0.515 |
| original_firstorder_Range.1 | 0.514 |
| original_glrlm_RunEntropy.3.1 | 0.514 |
| original_firstorder_RootMeanSquared.1st–2nd | 0.514 |
| original_glrlm_LongRunHighGrayLevelEmphasis.1st–2nd | 0.514 |
| original_shape_MajorAxisLength.4 | 0.513 |
| original_gldm_DependenceEntropy.4 | 0.513 |
| original_gldm_LargeDependenceHighGrayLevelEmphasis.4 | 0.513 |
| original_glrlm_LongRunHighGrayLevelEmphasis.2 | 0.513 |
| original_glcm_DifferenceEntropy.2nd–4th | 0.513 |
| original_firstorder_10Percentile.2 | 0.513 |
| original_firstorder_90Percentile.2nd–3rd | 0.513 |
| original_firstorder_Kurtosis.2nd–3rd | 0.513 |
| original_glcm_Autocorrelation.2nd–3rd | 0.513 |
| original_glcm_JointEnergy.2nd–3rd | 0.513 |
| original_glcm_MCC.2nd–4th | 0.513 |
| original_shape_Sphericity.3rd–4th | 0.513 |
| original_glcm_Autocorrelation.3rd–4th | 0.513 |
| original_glrlm_LongRunLowGrayLevelEmphasis.3rd–4th | 0.513 |
| original_glszm_SmallAreaLowGrayLevelEmphasis.3rd–4th | 0.513 |
| original_glszm_LowGrayLevelZoneEmphasis.3 | 0.512 |
| original_glrlm_RunLengthNonUniformity.4 | 0.512 |
| original_shape_Maximum2DDiameterSlice.4 | 0.511 |
| original_shape_Maximum3DDiameter.4 | 0.511 |
| original_firstorder_MeanAbsoluteDeviation.2 | 0.510 |
| original_glrlm_LongRunLowGrayLevelEmphasis.2 | 0.510 |
| original_glcm_MaximumProbability.2nd–4th | 0.510 |
| original_shape_Maximum2DDiameterSlice.1st–2nd | 0.509 |
| original_glcm_Contrast.4.1 | 0.509 |
| original_gldm_DependenceNonUniformity.4 | 0.507 |
| original_firstorder_Mean.3.1 | 0.507 |
| original_glcm_ClusterProminence.3.1 | 0.507 |
| original_glcm_JointAverage.2nd–3rd | 0.506 |
| original_glcm_JointEntropy.2nd–3rd | 0.506 |
| original_glcm_SumAverage.2nd–3rd | 0.506 |
| original_shape_MinorAxisLength.3rd–4th | 0.506 |
| original_glcm_JointAverage.3rd–4th | 0.506 |
| original_glcm_SumAverage.3rd–4th | 0.506 |
| original_glcm_SumEntropy.3rd–4th | 0.506 |
| original_gldm_DependenceNonUniformityNormalized.3rd–4th | 0.506 |
| original_gldm_LowGrayLevelEmphasis.3rd–4th | 0.506 |
| original_gldm_SmallDependenceLowGrayLevelEmphasis.3rd–4th | 0.506 |
| original_glrlm_LowGrayLevelRunEmphasis.3rd–4th | 0.506 |
| original_glrlm_ShortRunLowGrayLevelEmphasis.3rd–4th | 0.506 |
| original_shape_Elongation.3 | 0.506 |
| original_gldm_DependenceNonUniformity.3 | 0.506 |
| original_glszm_SizeZoneNonUniformity.3 | 0.506 |
| original_glcm_MCC.4.1 | 0.506 |
| original_firstorder_RobustMeanAbsoluteDeviation.2 | 0.505 |
| original_glcm_Autocorrelation.1st–2nd | 0.505 |
| original_glszm_SmallAreaLowGrayLevelEmphasis.1st–2nd | 0.505 |
| original_shape_Maximum2DDiameterRow.4.1 | 0.503 |
| original_glcm_Autocorrelation.4.1 | 0.503 |
| original_glcm_Id.4.1 | 0.503 |
| original_glszm_HighGrayLevelZoneEmphasis.4.1 | 0.503 |
| original_gldm_GrayLevelVariance.2 | 0.503 |
| original_glcm_SumEntropy.2nd–4th | 0.503 |
| original_glrlm_ShortRunLowGrayLevelEmphasis.1 | 0.502 |
| original_glszm_ZoneEntropy.1 | 0.502 |
| original_glrlm_LowGrayLevelRunEmphasis.1 | 0.500 |
| original_glrlm_GrayLevelVariance.2 | 0.500 |
| original_firstorder_10Percentile.3 | 0.500 |
| original_glcm_DifferenceEntropy.4 | 0.500 |
| original_glcm_DifferenceEntropy.3.1 | 0.500 |
| original_glcm_InverseVariance.3.1 | 0.500 |
| original_gldm_DependenceEntropy.3.1 | 0.500 |
| original_gldm_SmallDependenceHighGrayLevelEmphasis.3.1 | 0.500 |
| original_glszm_ZoneEntropy.3.1 | 0.500 |
| original_glcm_DifferenceAverage.4.1 | 0.500 |
| original_gldm_SmallDependenceEmphasis.2nd–3rd | 0.500 |
| original_firstorder_RobustMeanAbsoluteDeviation.2nd–4th | 0.500 |
| original_firstorder_90Percentile.3rd–4th | 0.500 |
| original_gldm_LargeDependenceLowGrayLevelEmphasis.3rd–4th | 0.500 |
| original_glszm_LargeAreaLowGrayLevelEmphasis.3rd–4th | 0.500 |
| original_glszm_ZoneEntropy.3rd–4th | 0.500 |
| original_glszm_SmallAreaHighGrayLevelEmphasis.4 | 0.499 |
| original_gldm_LowGrayLevelEmphasis.1 | 0.498 |
| original_gldm_LowGrayLevelEmphasis.2 | 0.498 |
| original_shape_Sphericity.2nd–4th | 0.498 |
| original_glcm_Idn.4 | 0.497 |
| original_gldm_LargeDependenceLowGrayLevelEmphasis.4.1 | 0.497 |
| original_glcm_InverseVariance.4 | 0.496 |
| original_gldm_HighGrayLevelEmphasis.2 | 0.495 |
| original_glrlm_HighGrayLevelRunEmphasis.2 | 0.495 |
| original_glcm_Idmn.2nd–4th | 0.495 |
| original_ngtdm_Contrast.4.1 | 0.494 |
| original_glcm_JointAverage.4.1 | 0.494 |
| original_glcm_SumAverage.4.1 | 0.494 |
| original_ngtdm_Busyness.2nd–3rd | 0.494 |
| original_firstorder_RootMeanSquared.3rd–4th | 0.494 |
| original_ngtdm_Complexity.3.1 | 0.493 |
| original_gldm_SmallDependenceLowGrayLevelEmphasis.4 | 0.493 |
| original_firstorder_Variance.2 | 0.493 |
| original_glcm_Autocorrelation.2 | 0.493 |
| original_glrlm_LongRunHighGrayLevelEmphasis.4.1 | 0.491 |
| original_shape_MajorAxisLength.1st–2nd | 0.491 |
| original_gldm_HighGrayLevelEmphasis.1st–2nd | 0.491 |
| original_glrlm_LowGrayLevelRunEmphasis.2 | 0.490 |
| original_glrlm_ShortRunHighGrayLevelEmphasis.2 | 0.490 |
| original_glrlm_ShortRunLowGrayLevelEmphasis.2 | 0.490 |
| original_glcm_Idm.4.1 | 0.488 |
| original_glrlm_LongRunLowGrayLevelEmphasis.4.1 | 0.488 |
| original_glszm_SmallAreaHighGrayLevelEmphasis.2nd–4th | 0.488 |
| original_glcm_DifferenceEntropy.2 | 0.488 |
| original_firstorder_Mean.3rd–4th | 0.488 |
| original_glszm_LowGrayLevelZoneEmphasis.3rd–4th | 0.488 |
| original_shape_Maximum2DDiameterColumn.4 | 0.487 |
| original_firstorder_Mean.1st–2nd | 0.486 |
| original_glszm_HighGrayLevelZoneEmphasis.1st–2nd | 0.486 |
| original_shape_Maximum3DDiameter.3.1 | 0.486 |
| original_glcm_DifferenceVariance.3.1 | 0.486 |
| original_glszm_LowGrayLevelZoneEmphasis.1 | 0.486 |
| original_glszm_SmallAreaEmphasis.1 | 0.486 |
| original_shape_Elongation.4.1 | 0.485 |
| original_gldm_LowGrayLevelEmphasis.4.1 | 0.485 |
| original_glrlm_LowGrayLevelRunEmphasis.4.1 | 0.485 |
| original_glcm_JointAverage.2 | 0.485 |
| original_glcm_SumAverage.2 | 0.485 |
| original_shape_Elongation.2 | 0.485 |
| original_glcm_Correlation.2nd–4th | 0.483 |
| original_glcm_Idmn.3 | 0.482 |
| original_glszm_ZoneEntropy.3 | 0.482 |
| original_shape_MajorAxisLength.4.1 | 0.482 |
| original_glrlm_HighGrayLevelRunEmphasis.1st–2nd | 0.482 |
| original_glszm_SmallAreaHighGrayLevelEmphasis.1st–2nd | 0.482 |
| original_firstorder_Skewness.3rd–4th | 0.481 |
| original_firstorder_TotalEnergy.4 | 0.481 |
| original_firstorder_10Percentile.1 | 0.481 |
| original_shape_Sphericity.1 | 0.481 |
| original_glszm_SizeZoneNonUniformity.4 | 0.479 |
| original_glcm_Idmn.4.1 | 0.479 |
| original_glszm_GrayLevelVariance.3.1 | 0.479 |
| original_glszm_LowGrayLevelZoneEmphasis.3.1 | 0.479 |
| original_glszm_SizeZoneNonUniformityNormalized.1 | 0.478 |
| original_glcm_DifferenceAverage.2nd–4th | 0.478 |
| original_glrlm_ShortRunHighGrayLevelEmphasis.1st–2nd | 0.477 |
| original_gldm_SmallDependenceHighGrayLevelEmphasis.4.1 | 0.476 |
| original_gldm_LargeDependenceHighGrayLevelEmphasis.3 | 0.476 |
| original_gldm_SmallDependenceLowGrayLevelEmphasis.4.1 | 0.476 |
| original_glcm_MCC.2nd–3rd | 0.475 |
| original_firstorder_Maximum.3rd–4th | 0.475 |
| original_glrlm_RunVariance.2nd–4th | 0.473 |
| original_glcm_Autocorrelation.3.1 | 0.472 |
| original_glcm_JointAverage.3.1 | 0.472 |
| original_glcm_SumAverage.3.1 | 0.472 |
| original_glcm_Idn.3 | 0.471 |
| original_glrlm_ShortRunLowGrayLevelEmphasis.4.1 | 0.471 |
| original_shape_Maximum2DDiameterSlice.3rd–4th | 0.469 |
| original_glcm_Correlation.3rd–4th | 0.469 |
| original_glszm_SmallAreaEmphasis.3rd–4th | 0.469 |
| original_glcm_MCC.1 | 0.466 |
| original_glrlm_ShortRunHighGrayLevelEmphasis.3.1 | 0.465 |
| original_glszm_GrayLevelNonUniformityNormalized.3.1 | 0.465 |
| original_glcm_SumSquares.2nd–4th | 0.465 |
| original_glszm_LargeAreaHighGrayLevelEmphasis.4 | 0.465 |
| original_firstorder_Energy.3 | 0.465 |
| original_firstorder_TotalEnergy.3 | 0.465 |
| original_shape_Sphericity.4.1 | 0.465 |
| original_glcm_JointEnergy.3rd–4th | 0.463 |
| original_firstorder_Minimum.1st–2nd | 0.461 |
| original_ngtdm_Busyness.4.1 | 0.459 |
| original_gldm_HighGrayLevelEmphasis.3.1 | 0.458 |
| original_firstorder_10Percentile.3rd–4th | 0.456 |
| original_firstorder_InterquartileRange.4 | 0.456 |
| original_firstorder_Range.4.1 | 0.453 |
| original_glrlm_HighGrayLevelRunEmphasis.3.1 | 0.451 |
| original_glcm_DifferenceVariance.2 | 0.450 |
| original_firstorder_90Percentile.1st–2nd | 0.450 |
| original_glszm_LargeAreaLowGrayLevelEmphasis.1st–2nd | 0.450 |
| original_glcm_SumEntropy.2nd–3rd | 0.450 |
| original_gldm_SmallDependenceHighGrayLevelEmphasis.2 | 0.448 |
| original_ngtdm_Complexity.4 | 0.447 |
| original_ngtdm_Complexity.1 | 0.444 |
| original_firstorder_Variance.4.1 | 0.444 |
| original_glcm_InverseVariance.4.1 | 0.444 |
| original_gldm_GrayLevelVariance.4.1 | 0.444 |
| original_glrlm_GrayLevelVariance.4.1 | 0.444 |
| original_shape_Sphericity.4 | 0.443 |
| original_glrlm_LongRunHighGrayLevelEmphasis.3.1 | 0.438 |
| original_glrlm_RunVariance.2nd–3rd | 0.438 |
| original_firstorder_Minimum.3rd–4th | 0.438 |
| original_firstorder_MeanAbsoluteDeviation.4.1 | 0.432 |
| original_glcm_Imc1.1st–4th | 0.432 |
| original_firstorder_Kurtosis.3rd–4th | 0.431 |
| original_firstorder_Skewness.3 | 0.429 |
| original_shape_Maximum2DDiameterRow.3rd–4th | 0.425 |
| original_gldm_DependenceEntropy.3rd–4th | 0.425 |
| original_firstorder_Skewness.3.1 | 0.424 |
| original_glszm_SmallAreaEmphasis.2nd–3rd | 0.419 |
| original_shape_Maximum3DDiameter.3 | 0.412 |
| original_firstorder_Minimum.4.1 | 0.363 |

*Abbreviations:* AUC = area under the curve; glcm = Grey level co-occurrence matrix; ngtdm = neighbourhood grey tone difference matrix; glrlm =Grey level run length matrix; ngtdm =Neighbourhood grey tone difference matrix; glszm =Gray Level Size Zone Matrix;
