# Supplementary material for: Prognosis prediction of uterine cervical cancer using changes in the histogram and texture features of apparent diffusion coefficient during definitive chemoradiotherapy
Source: PLoS One. 2023 Mar 31;18(3):e0282710. doi: 10.1371/journal.pone.0282710 (PMC10065283; doi:10.1371/journal.pone.0282710)
Supplement: Attachment — Submitted filename: ADCchange_manuscript_plos_ZJreviewed.docx [file pone.0282710.s007.docx]

**Title**

**Prognosis prediction of uterine cervical cancer using changes in the histogram and texture features of apparent diffusion coefficient during definitive chemoradiotherapy**

Akiyo Takada^1^*, Hajime Yokota^2^, Miho Watanabe Nemoto^2^, Takuro Horikoshi^1^, Koji Matsumoto^1^, Yuji Habu^3^, Hirokazu Usui^3^, Katsuhiro Nasu^1^, Makio Shozu^3^, Takashi Uno^2^

*1. Radiology, Department of Radiology, Chiba University Hospital, Chiba, Japan*

*2. Radiology, Department of Diagnostic Radiology and Radiation Oncology, Graduate School of Medicine, Chiba University, Chiba, Japan*

*3. Obstetrics and gynecology, Department of Reproductive Medicine, Graduate School of Medicine, Chiba University, Chiba, Japan*

* Corresponding author

E-mail: [r10.aa.0826@gmail.com](mailto:r10.aa.0826@gmail.com) (AT)

**Abstract**

**Objectives** We investigated prospectively whether, in cervical cancer (CC) treated with concurrent chemoradiotherapy (CCRT), the Apparent diffusion coefficient (ADC) histogram and texture parameters and their change rates during treatment could predict prognosis.

**Methods** Fifty-seven CC patients treated with CCRT at our institution were included. They underwent up to four times MRI scans during the treatment course (1st: before treatment [n=41], 2nd: at the start of image-guided brachytherapy (IGBT) [n=41], 3rd: in the middle of IGBT [n=27], 4th: after treatment [n=53]). The entire tumour was set as the volume of interest (VOI) manually in the axial images of the ADC map by two radiologists. A total of 107 image features (morphology features 14, histogram features 18, texture features 75) were extracted from the VOI. The recurrence prediction values of the features and their change rates were evaluated by Receiver operating characteristics (ROC) analysis. The presence or absence of local and distant recurrence within two years was set as an outcome. The intraclass correlation coefficient (ICC) was also calculated.

**Results** The change rates in kurtosis between the 1^st^ and 3^rd^, and 1^st^ and 2^nd^ MRIs, and the change rate in grey level co-occurrence matrix_cluster shade between the 2^nd^ and 3^rd^ MRIs showed particularly high predictive powers (Area under the ROC curve = 0.784, 0.759, and 0.750, respectively) , which exceeded predictive abilities of the parameters obtained from pre-treatment or post-treatment MRI only. The change rate in kurtosis between the 1^st^ and 2^nd^ MRIs had good reliability (ICC = 0.765).

**Conclusions** The change rate in ADC kurtosis between the 1^st^ and 2^nd^ MRIs was the most reliable parameter, enabling us to predict prognosis early in the treatment course.

**Introduction**

Uterine cervical cancer (CC) is among the most commonly diagnosed cancers in women, resulting in over 300,000 deaths annually worldwide [1]. For International Federation of Gynecology and Obstetrics (FIGO) stage 1B to 4A, concurrent chemoradiotherapy (CCRT) is the current standard of treatment. Image-guided brachytherapy (IGBT) planning has become available, and standard approaches have been developed [2]. In recent years, a treatment plan tailored according to the risk of recurrence and expected adverse events, so-called ‘precision medicine’, has become common. Three-dimensional (3D) IGBT is a new brachytherapy planning method for CCRT that uses 3D images acquired from MRI and CT. 3D-IGBT has achieved dose-distribution control according to the shape of individual tumours and has the potential to allow dose adjustment according to the recurrence risk [3]. Therefore, accurate recurrence risk prediction is essential to perform precision medicine with 3D-IGBT for CC.

Prognostic factors for CC have included age, race, FIGO stage, histological type and grade, tumour volume, lymph node involvement and location, lymphovascular space invasion, and performance status [1], but their accuracy is limited. There was no significant difference in FIGO stage (FIGO2008) and tumour volume between recurrent and non-recurrent disease in previous studies [4, 5]. 3D-IGBT improves prognosis for advanced bulky tumours [6, 7], decreasing the prognostic value of tumour volume. Lymph node involvement and lymphovascular space invasion are information that can only be obtained by surgical pathology, which is impossible for patients receiving CCRT. Therefore, prognostication using images has been attracting attention. In particular, the utility of MRI has been reported [8-10].

Several studies have reported the accuracy of apparent diffusion coefficient **(**ADC) values of the tumour in predicting lymph node metastasis. These values are also helpful in identifying an early response to CCRT, and in predicting mid- to long-term prognosis in CC [9, 11, 12]. Most of them use pretreatment MRI scans [9, 12]. We hypothesized that ADC histogram and texture features from MRIs during the treatment course and their change rates would reflect the response to treatment and might improve the prognostic accuracy. Arriving at a prognosis early in the clinical course would help in further treatment planning.

The purpose of this study was to investigate whether histogram and texture features of ADC and their change rates during CCRT could predict the disease course of CC in patients treated with CCRT.

**Materials and methods**

**1. Patients**

**1A. Enrolment**

The ethical committee of our institute approved this study and informed consent was obtained from each patient. We prospectively assessed 125 CC patients who underwent definitive CCRT between June 2015 and October 2019 at our institution (Fig 1). The patients were slated to undergo four MRI scans: one before starting treatment, one just before the start of IGBT, one in the middle of IGBT (at the end of external beam radiotherapy (EBRT)), and one 2–3 months after the end of treatment (Fig 2). These time points were determined in consultation with the radiation oncologists when they were considered important for observing the therapeutic effect. The following inclusion criteria were applied: (a) patients who underwent a 3T MRI (Ingenia, Philips Medical Systems, Best, Netherland) at least once of four time points (b) the primary tumour was identified in the pretreatment MRI (c) patients with ≧2 years of follow-up. A total of 57 patients matched the criteria. (Fig 1).

**Fig 1. Flowchart showing the patient enrolment and inclusion criteria.**

The numbers of MR examinations available at each time point and the number of calculated change rates are shown.

**Fig 2. Flowchart of treatment, MRI acquisition, and imaging processing.**

CCRT = cervical cancer radiation therapy; IGBT = image-guided brachytherapy; EBRT = External beam radiotherapy; HR-CTV = high-risk clinical target volume; CDDP = cisplatin

**1B. Treatment**

Treatment strategy was determined in consultation with gynaecologists and radiation oncologists according to each patient’s clinical status, clinical stage, and histological type. Our institute's standard treatment protocol for CC is the same for squamous cell carcinoma (SqCC), adenocarcinoma, and adenosquamous carcinoma. The primary tumour and regional lymph nodes at risk are treated with EBRT at a dose of 50 Gy (2.0 Gy per fraction) combined with concurrent daily cisplatin chemotherapy [13]. The high-risk clinical target volume (CTV) [14], determined after EBRT of 30 Gy [15] was then treated with 18–24 Gy 3D-IGBT (Fig 2).

**1C. Follow-up**

The patients were followed up by gynecologists and radiation oncologists. In the first two years, pelvic examinations were performed, and cytology and tumour markers (CEA, CA19-9, CA125, SCC antigen) were acquired, every 2–3 months. Follow-up CT was performed three months after treatment completion and annually for two years. When recurrence was suspected with the above examinations, additional CT, MRI (not counted as a time point), or PET was performed.

**2. Imaging**
**2A. MRI acquisition**

The standardized image acquisition protocol included T2-weighted images (T2WI) T1-weighted images (T1WI), and contrast-enhanced T1WI in the axial and sagittal planes. Diffusion-weighted images (DWI) were acquired in the axial plane. The main scan parameters are shown in Table 1. All MRIs were acquired without brachytherapy devices.

**Table 1. Summary of MRI acquisition protocols.**

| Sequences | T2WI | T2WI | DWI | CE-T1WI |
| --- | --- | --- | --- | --- |
| Plane | Sagittal | axial | axial | sagittal |
| TR/TE | 3000/100 | 3000/100 | 5000/90 | 540/15 |
| Field of view(mm) | 240 × 240 | 240 × 240 | 230 × 230 | 240 × 240 |
| Matrix size | 480 × 480 | 704 × 704 | 128 × 128 | 384 × 384 |
| Slice thickness (mm) | 5 | 4 | 5 | 5 |
| Intersection gap (mm) | 1 | 1 | 1 | 1 |
| Number of excitations | 1 | 1 | 2 | 2 |
| b value (sec/mm^2^) |  |  | 1000 |  |

*Abbreviations: rec* = recurrent, non-rec= non-recurrent

**2B. Segmentation**

The entire tumour was set as the volume of interest (VOI) in the axial images of the ADC map by a radiologist (10 years' experience in pelvic MRI), using T2WI, contrast-enhanced T1WI, and pelvic examination findings for guidance. Non-enhanced areas which considered to be cystic, necrotic and/or hemorrhagic were excluded. If no residual tumour was observed after treatment, the VOI was placed in the area where the lesion had been on the previous MRI. To examine inter-observer reliability, a second radiologist with 15 years’ experience in pelvic MRI independently set VOIs on 96 MRIs (24 MRIs per time point) of 24 cases from those patients that had undergone four MRIs of all time points during the treatment course.

**2C. Feature extraction**

A total of 107 image features (morphology features 14, histogram features 18, texture features 75) were extracted from the VOIs using open-source software, PyRadiomics v3.0 [16, 17]. Differences between PyRadiomics and the Image Biomarker Standardization Initiative (IBSI) have already been examined and are in agreement [18].

An absolute rescaling method (0–4 × 10^-3^mm^2^/sec) was applied to normalise ADC values. Pixel values between the upper and lower limits were resampled into 64 levels.

The change rates in the image features were calculated as follows:

$Change rate = (later feature value - previous feature value)/previous feature value$(1)

We calculated the change rates from six combinations of two MRI examinations (between 1^st^ and 2^nd^, 1^st^ and 3^rd^, 1^st^ and 4^th^, 2^nd^ and 3^rd^, 2^nd^ and 4^th^, 3^rd^ and 4^th^). We refer to the image features from 1^st^, 2^nd^, 3^rd^ and 4^th^ MRIs and their change rates as ‘image parameters’.

**3. Statistics**
**3A. Univariate analysis**

Univariate analyses were performed on clinical data such as age, FIGO stage (FIGO2008), and histological type using the Fisher’s exact and Mann–Whitney tests. Image parameters were compared with the Mann–Whitney test.

**3B. Receiver operating characteristic (ROC) and survival analyses**

In the 57 patients, the recurrence prediction ability of the image parameters was evaluated by ROC analysis. The presence or absence of local and/or distant recurrence within the 2 years was set as an outcome. No patients were lost to follow-up. The cut-off values were determined from the ROC curve using the maximum Youden index for image parameters, which showed a high area under the curve (AUC). Kaplan-Meier plots of disease-free survival (DFS) were constructed with the cut-off value, and the log-rank test was used for comparison. We also performed the same ROC analysis only in patients with SqCC, calculated cut-off values, and constructed Kaplan-Meier plots in the same way.

**3C. Inter-observer reliability**

The Dice similarity coefficient (DSC), used to indicate the overlap ratio between the VOIs drawn by two radiologists, was calculated to compare all sets of VOIs. The intraclass correlation coefficient (ICC) was also calculated for the image parameters with high AUCs in the ROC. DSC scores > 0.70 are generally considered as having good agreement [19]. The ICC values were interpreted as follows: “poor” (ICC < 0.5), "moderate" (0.5–0.75), “good” (0.75–0.9) and "excellent" (ICC > 0.9) [20, 21].

**Results**

**1. Eligible MRIs**

We only included MRIs taken by the 3T MRI (Ingenia, Philips Medical Systems, Best, Netherland). For 57 patients, 41, 41, 27, and 53 MRIs were available at time points 1, 2, 3, and 4, respectively. (Fig 1). The change rates from six combinations of two MRI examinations (between 1^st^ and 2^nd^, 1^st^ and 3^rd^, 1^st^ and 4^th^, 2^nd^ and 3^rd^, 2^nd^ and 4^th^, 3^rd^ and 4^th^) were calculated as shown in Fig 1.

**2. Patients’ clinical characteristics**

Twenty-three of 57 patients had developed a recurrence within two years and 34 patients had had no recurrence within two years of follow-up. In 23 patients with recurrence, eight patients had local pelvic recurrence (cervix and lymph nodes) within the irradiation field, and 15 patients had developed distant metastasis. Clinical data and the results of the univariate analyses are shown in Table 2. Forty-nine of the 57 patients had SqCC. The results of the analysis in SqCC patients only are shown in the supplemental materials (S1 and S2 Tables, S1 Fig). The histological type showed a significant difference (*p* = 0.014), with adenocarcinoma recurring more frequently than the other types. The FIGO stage showed no significant difference between the recurrent group and the non-recurrence group.

**Table 2. Clinical data and the results of the univariate analyses.**

|  |  | All (n=57) | Rec (n=23) | Non-rec (n=34) | *p* |
| --- | --- | --- | --- | --- | --- |
| Site of recurrence | local |  | 8 |  |  |
|  | distant |  | 15 |  |  |
| Age  (mean ± standard deviation) |  | 61 ± 14.3 | 60.6 ± 13.7 | 61.3 ± 14.7 | 0.839 |
| FIGO | 1B | 15 | 5 | 10 | 0.659 |
|  | 2A | 10 | 5 | 5 |  |
|  | 2B | 14 | 5 | 9 |  |
|  | 3A | 2 | 1 | 1 |  |
|  | 3B | 12 | 5 | 7 |  |
|  | 4A | 4 | 2 | 2 |  |
| Pathological type | SqCC | 49 | 17 | 32 | 0.014 |
|  | Adenocarcinoma | 7 | 6 | 1 |  |
|  | Adenosquamous | 1 | 0 | 1 |  |

**3. ROC analysis**

**3A. Summary of ROC analysis**

ROC analysis was performed in the 57 cases; the image parameters with high AUCs are shown in Table 3. The predictive power of the change rates in kurtosis (sharpness of the histogram peaks) between the 1^st^ and 3^rd^, and 1^st^ and 2^nd^ scans, and the change rate in grey level co-occurrence matrix (glcm)_cluster shade between the 2^nd^ and 3^rd^ scans, were particularly high (AUC = 0.784, 0.759, and 0.750, respectively). Among the image features of the 1^st^ MRI, glcm_Inverse Difference had the best prognostic accuracy (AUC = 0.737). The AUCs of the all image parameters are described in the Supplemental Materials (S3 Table).

**Table 3. The image parameters with high AUCs in all patients.**

| **image parameters** | **status** | **AUC** |
| --- | --- | --- |
| Kurtosis. | change rate 1^st^–3^rd^ | 0.785 |
| Kurtosis. | change rate 1^st^–2^nd^ | 0.759 |
| glcm_ClusterShade. | change rate 2^nd^–3^rd^ | 0.750 |
| glcm_Id. | 1^st^ | 0.737 |
| glcm_Imc2. | change rate 1^st^–3^rd^ | 0.736 |
| glcm_Idm. | 1^st^ | 0.734 |
| gldm_DependenceVariance. | 1^st^ | 0.734 |
| shape_SurfaceVolumeRatio. | 2^nd^ | 0.733 |
| gldm_DependenceNonUniformityNormalized. | 1^st^ | 0.732 |
| glszm_LargeAreaHighGrayLevelEmphasis. | change rate 1^st^–3^rd^ | 0.729 |

*Abbreviations:* glcm = Grey level co-occurrence matrix; glcm_Id= Grey level co-occurrence matrix Inverse Difference; glcm_imc2 = Grey level co-occurrence matrix informational measure of correlation; gldm= Gray Level Dependence Matrix; glszm = Gray Level Size Zone Matrix

**3B. Morphology parameters**

The AUC of shape_VoxelVolume on the 1st MRI (pretreatment tumour volume) as a conventional prognostic factor was 0.614. The change rate in shape_VoxelVolume between the 1^st^ and 4^th^ MRI, which represents changes in tumour volume before and after treatment, showed an AUC of 0.600.

**3C. Histogram parameters**

Among the histogram parameters, the change rates in kurtosis between the 1^st^ and 3^rd^ MRIs (kurtosis change 1^st^ –3^rd^) and between the 1^st^ and 2^nd^ MRIs (kurtosis change 1^st^ –2^nd^) showed high AUCs (0.784 and 0.759): the highest and the second highest of all parameters. The kurtosis change 1^st^ –2^nd^ and 1^st^ –3^rd^ were significantly lower in the recurrence group than in the nonrecurrence group (*p* = 0.018 and 0.020, respectively); that is, kurtosis showed a greater decrease between the 1^st^ and the 2^nd^ or 3^rd^ MRI in the recurrence group. (Table 4).

**Table 4. The mean and standard deviation (SD) of kurtosis at each time point in the recurrent and non-recurrent groups.**

|  | Non-recurrent | | reccurent | |
| --- | --- | --- | --- | --- |
|  | mean | SD | mean | SD |
| kurtosis change 1^st^ – 2^nd^ | −0.036 | 0.310 | −0.317 | 0.153 |
| kurtosis change 1^st^ – 3^rd^ | −0.126 | 0.654 | −0.452 | 0.171 |
| glcm_ClusterShade change 2^nd^ – 3^rd^ | −18.682 | 40.515 | −1.143 | 0.498 |

**3D. Texture parameters**

Among the texture parameters, the change rate in glcm_ClusterShade between the 2^nd^ and 3^rd^ MRIs (glcm_ClusterShade change 2^nd^ –3^rd^) showed the highest AUC (0.750): the third highest of all parameters. The glcm_ClusterShade change 2^nd^ –3^rd^ was significantly higher in the recurrence group than in the nonrecurrence group (*p* = 0.036) (Table 4).

**4. Survival analysis**

We determined the cut-off values for the three parameters with high AUCs (kurtosis change 1^st^ –3^rd^, kurtosis change 1^st^ –2^nd^, and glcm_ClusterShade change 2^nd^ –3^rd^). Kaplan-Meier plots were created with the cut-off values (Fig 3). Each parameter divided the patients with better and worse prognoses (*p* = 0.032, 0.0087, and 0.008, respectively). Box-and-whisker plots of kurtosis in the 1^st^ to 4^th^ MRI in the recurrent group and the nonrecurrent group are shown in Fig 4.

**Fig 3.** **Kaplan-Meier plots for all patients. Log-rank *p* values were calculated.**

Glcm = Grey level co-occurrence matrix

**Fig 4. Box-and-whisker plots of ADC kurtosis**

Box-and-whisker plots shows trends of ADC kurtosis during the clinical course in the recurrent and non-recurrent groups. The mean value of kurtosis is indicated as the × symbol.

ADC kurtosis tended to decrease towards the end of treatment as an overall trend in both groups. The decrease in kurtosis from the first to the third MRI was greater in the recurrent group than in the non-recurrent group.

**5. Inter-observer reliability**

The mean DSC scores for the VOIs of each imaging time point are shown in Table 5-a. ICC scores of kurtosis and glcm_ClusterShade at each imaging time point and the three image parameters are shown in Table 5-b. DSCs of the 1st and 2nd MRI were good (0.852 and 0.763), while DSCs of the 3rd and 4th MRI were poor (0.430 and 0.438). Kurtosis of the 1st and 2nd MRI and the kurtosis change 1^st^ –2^nd^ had moderate-to-good reliability (ICC = 0.783, 0.729, and 0.765, respectively), while kurtosis of the 3^rd^ MRI and the kurtosis change 1^st^ –3^rd^ had poor reliability (ICC = 0.222 and 0.408). Glcm_ClusterShade of 2nd and 3rd MRIs and the glcm_ClusterShade change 2^nd^ –3^rd^ had poor reliability (ICC ≤ 0.001, 0.106, and < 0.001, respectively).

**Table 5-a. Dice similarity coefficient (DSC) scores (average and standard deviation) in 1st–4th time points.**

|  | 1^st^ | 2^nd^ | 3^rd^ | 4^th^ |
| --- | --- | --- | --- | --- |
| mean | 0.852 | 0.763 | 0.43 | 0.438 |
| SD | 0.073 | 0.164 | 0.291 | 0.242 |

**Table 5-b. Intraclass correlation coefficients (ICC) values of the ADC kurtosis and the glcm_ClusterShade in 1st–4th time points.**

|  | 1^st^ | 2^nd^ | 3^rd^ | 4^th^ | change rate  1^st^–2^nd^ | change rate  1^st^–3^rd^ | change rate  2^nd^–3^rd^ |
| --- | --- | --- | --- | --- | --- | --- | --- |
| kurtosis | 0.783 | 0.729 | 0.222 | 0 | 0.765 | 0.408 | ― |
| glcm_ClusterShade | 0.714 | < 0.001 | 0.106 | 0.580 | ― | ― | < 0.001 |

*Abbreviations:* glcm = Grey level co-occurrence matrix

**Discussion**

Image features of ADC values and their change rates during the disease course and treatment may predict the prognosis with high accuracy in CC patients. The predictive power of the kurtosis changes 1^st^ –3^rd^ and 1^st^ –2^nd^ and the glcm_ClusterShade change 2^nd^ –3^rd^ were particularly high (AUC ≥ 0.75). Prognostic prediction abilities of these change rates were higher than the parameters obtained from pretreatment or posttreatment MRI only. These three change rates also showed high predictive accuracy in the analysis of the SqCC patients. The kurtosis change 1^st^ –2^nd^ was considered the most reliable because the kurtosis of the 1^st^ and 2^nd^ MRIs and the kurtosis changes between them showed high ICC scores.

Kurtosis reflects the degree of sharpness of the histogram and the combined weight of the histogram tails relative to the rest of the distribution. The kurtosis increases as the peak becomes higher and the tails become heavier, while it decreases as the peak becomes flatter and the tails become lighter. A high peak suggests homogeneity, and a flat peak suggests variability or heterogeneity [22]. Lower tumour ADC kurtosis may indicate heterogeneity of the tumour ADC values, although histogram tails or outliers somehow affect kurtosis [5, 23]. Ciolina et al. reported that lower tumour ADC kurtosis in pretreatment MRI might reflect more disorganized tumour tissues with ‘nuclear enlargement and an ineffective vascular net’, which reduced the efficacy of chemotherapy in CC [5]. Shaghaghi et al. reported that lower tumour kurtosis represents an increase in tissue heterogeneity and scattered necrosis within the tumour in HCC treated with transcatheter arterial chemoembolization [24]. During treatment, a decrease in tumour ADC probably reflects therapy-induced heterogeneous changes, which may include scattered necrosis, destroyed tumour vasculature, and nonuniform tumour cell morphologies.

In our study, ADC kurtosis tended to decrease more in the recurrent group than in the nonrecurrent group, particularly between the 1^st^ and 2^nd^ or 3^rd^ MRI (Fig 4). Increased intratumoural necrotic and hypoxic tissue cause resistance for radiation therapy [25]. Therefore, a greater decrease in tumour ADC kurtosis between the 1^st^ and 2^nd^ /3^rd^ MRI, representing extensive heterogenic and scattered necrotic changes within the tumour early during CCRT, could be a predictor of poor prognosis for CC treated with CCRT. Examples of ADC histograms of recurrent and non-recurrent cases are shown in Fig 5. Contrary to kurtosis, glcm_ClusterShade, a measure of the skewness and uniformity of the GLCM, tended to decrease less markedly between the 2^nd^ and 3^rd^ MRI in the recurrent group. A higher Glcm_ClusterShade implies greater asymmetry about the mean [26]. A higher Glcm_ClusterShade may also reflect heterogeneity of the tumour. Therefore, a small decrease in Glcm_ClusterShade might predict a poor prognosis.

**Fig 5-a. Recurrent case**

A 66-year-old patient with FIGO stage IIB squamous cell carcinoma. She recurred with distance lymph node metastasis in a year. An intra-tumoural T2WI hypointense area (arrow) with poor enhancement on T1WI was presumed to be necrosis. The necrotic area expanded on the following 2^nd^ and 3^rd^ scans. Although segmentation was performed to exclude necrosis, the decrease in kurtosis is conspicuous in the 2^nd^ and 3^rd^ scans. The sharpness of the histogram also decreases in the 2^nd^ and 3^rd^ scans, indicating decreasing kurtosis.

**Fig 5-b. Non-recurrent case**

A 70-year-old patient with FIGO stage IIB squamous cell carcinoma. She had been followed up for more than 2 years without recurrence. No obvious necrosis was observed in the tumour in the 2^nd^ and 3^rd^ scans. Kurtosis did not decrease, and there was no obvious decrease in histogram sharpness.

The ICCs of kurtosis tended to decline as treatment progressed, and were significantly associated with the shrinkage or disappearance of the tumour. When the tumour had disappeared, the VOI setting where the lesion was located at the previous MRI is subjective. A past study also reported that smaller tumour volumes contributed to poor inter-observer reliability of ADC [27]. The effects associated with gas in the intestine or in areas of necrosis should be more significant in small lesions than in large lesions. It may be improved by administration of scopolamine butylbromide before MRI acquisition and taking care not to include air during segmentation. Another cause is that the therapeutic effects obscure the boundary between the residual tumour and the surrounding normal tissue, making tumour segmentation difficult [28, 29]. The DSC scores were also lower at the 3^rd^ and 4^t^ than at the 1^st^ and 2^nd^ MRIs. Because of the high ICC, the kurtosis change 1^st^–2^nd^ is an excellent parameter with high predictive accuracy and reliability.

There have been a few studies predicting long-term prognosis using intra-treatment MRI, and the results were different from ours [8, 30, 31]. Past studies reported that changes in mean tumour ADC during treatment and energy on ADC maps of intra-treatment MRI showed high predictive power [8, 30]. In our study, mean intra-treatment ADC, energy, and their change rates, were not significantly different between the recurrent and nonrecurrent groups (AUC < 0.72, supplemental materials). In addition, the change rate in kurtosis was not useful in predicting prognosis in the past study [31]. These discrepancies may be due to the following factors: (1) differences in MR protocols and parameters, (2) different sample size, and (3) differences in the VOI placement method with or without non-enhanced areas within the tumour. A previous study that reported low predictability of kurtosis determined VOIs including cystic and necrotic areas within the tumor [31], although it is common to exclude non-enhanced areas to avoid outliers in imaging analyses for CC [8, 32, 33]. Kurtosis can be susceptible to error because it is affected not only by the degree of sharpness but also by the tails of the histogram. Air and haemorrhage in the necrotic tissue may prevent calculating accurate tumour ADC kurtosis.

Our study has some limitations. First, we had a relatively small study population and recurrent cases included both local and distant recurrence. However, our study has the largest number of patients among the papers about long-term prognosis prediction using change rates in ADC histogram and texture features during treatment for CC. Second, all patients had not been able to undergo MRI four times during the study, and the numbers of MRI available were different depending on the time point. The number of 3^rd^ MRIs was relatively small. Third, we could not pathologically confirm what the tumour ADC kurtosis reflected. Further study is needed to examine what the image parameters reflect pathologically in surgical specimens.

In conclusion, image features and their change rates in ADC during the treatment course were useful for predicting the prognosis after definitive CCRT for CC. They reflected the early response of the tumour tissue to CCRT; therefore, image parameters using intra-treatment MRI showed high predictive accuracy. The kurtosis change 1^st^–2^nd^ was the most reliable and has an advantage in predicting prognosis early during treatment before the initiation of IGBT. Predicting prognosis early during treatment enables the treating physicians to adjust the radiation and chemotherapy dose and to provide a more personalised treatment.

**Acknowledgments**

I have no acknowledgements.

**References**

1. Cohen PA, Jhingran A, Oaknin A, Denny L. Cervical cancer. Lancet. 2019;393:169–182. PMID: 30638582

2. Rijkmans EC, Nout RA, Rutten IHHM, Ketelaars M, Neelis KJ, Laman MS, et al. Improved survival of patients with cervical cancer treated with image-guided brachytherapy compared with conventional brachytherapy. Gynecol Oncol. 2014;135:231–238. PMID: 25172763

3. Okada K, Oike T, Ando K, Kubo N, Ohno T. Cervical Stump Cancer Treated With Radiotherapy Using Computed Tomography-Guided Brachytherapy. Cureus. 2021;13:3–7. PMID: 33842163

4. Takada A, Yokota H, Watanabe Nemoto M, Matsushima J, Uno T. A multi-scanner study of MRI radiomics in uterine cervical cancer: prediction of in-field tumor control after definitive radiotherapy based on a machine learning method including peritumoral regions. Jpn J Radiol. 2020;38:265–273. PMID: 31907716

5. Ciolina M, Vinci V, Villani L, Gigli S, Saldari M, Panici PB, et al. Texture analysis versus conventional MRI prognostic factors in predicting tumor response to neoadjuvant chemotherapy in patients with locally advanced cancer of the uterine cervix. Radiol Medica. 2019;124:955–964. PMID: 31254220

6. Pötter R, Tanderup K, Schmid MP, Jürgenliemk-Schulz I, Haie-Meder C, Fokdal LU, et al. MRI-guided adaptive brachytherapy in locally advanced cervical cancer (EMBRACE-I): a multicentre prospective cohort study. Lancet Oncol. 2021;22:538–547. PMID: 33794207

7. Sturdza A, Pötter R, Fokdal LU, Haie-Meder C, Tan LT, MAzeron R, et al. Image guided brachytherapy in locally advanced cervical cancer: Improved pelvic control and survival in RetroEMBRACE, a multicenter cohort study. Radiother Oncol. 2016;120:428–433. PMID: 27134181

8. Bae JM, Kim CK, Park JJ, Park BK. Can diffusion-weighted magnetic resonance imaging predict tumor recurrence of uterine cervical cancer after concurrent chemoradiotherapy ? Abdom Radiol. 2016;41:1604–1610. PMID: 27056747

9. Heo SH, Shin SS, Kim JW, Lim HS, Jeong YY, Kang WD, et al. Pre-Treatment Diffusion-Weighted MR Imaging for Predicting Tumor Recurrence in Uterine Cervical Cancer Treated with Concurrent Chemoradiation : Value of Histogram Analysis of Apparent Diffusion Coefficients. Korean J Radiol. 2013;14:616–625. PMID: 23901319

10. Gu K won, Kim CK, Choi CH, Yoon YC, Park W. Prognostic value of ADC quantification for clinical outcome in uterine cervical cancer treated with concurrent chemoradiotherapy. Eur Radiol. 2019;29:6236–6244. PMID: 30980126

11. Lee J, Kim CK, Park SY. Histogram analysis of apparent diffusion coefficients for predicting pelvic lymph node metastasis in patients with uterine cervical cancer. Magn Reson Mater Physics, Biol Med. 2020;33:283–292. PMID: 31549269

12. Wang M, Perucho JAU, Tse KY Chu MMY, Ip P, Lee EY. MRI texture features differentiate clinicopathological characteristics of cervical carcinoma. Eur Radiol. 2020; 30:5384–5391. PMID: 32382845

13. Hanawa S, Mitsuhashi A, Usui H, Yamamoto N, Watanabe-Nemoto M, Nishikimi K, et al. Daily low-dose Cisplatin-based concurrent chemoradiotherapy for the treatment of cervical cancer in patients 70 years or older. Int J Gynecol Cancer. 2015;25:891–6. PMID: 25811592

14. Pötter R, Haie-meder C, Limbergen EV, Barillot I, Brabandere MD, Dimopoulos J, et al. Recommendations from gynaecological ( GYN ) GEC ESTRO working group ( II ): Concepts and terms in 3D image-based treatment planning in cervix cancer brachytherapy — 3D dose volume parameters and aspects of 3D image-based anatomy, radiation physics, radiobiology. Radiother Oncol. 2006;78:67–77. PMID: 16403584

15) Ebina Y, Mikami M, Nagase S, Tabata T, Kaneuchi M, Tashiro H, et al. Japan Society of Gynecologic Oncology guidelines 2017 for the treatment of uterine cervical cancer. Int J Clin Oncol. 2019;24:1–19. PMID: 30291468

16. PY-RADIOMICS Open-source radiomics library written in python via <http://www.radiomics.io/pyradiomics.html> Accessed 7 May 2022.

17. Van Griethuysen JJM, Fedorov A, Parmar C, Hosny A, Aucoin N, Narayan V, et al. Computational radiomics system to decode the radiographic phenotype. Cancer Res. 2017; 77:e104–e107. PMID: 29092951

18. Zwanenburg A, Vallières M, Abdalah MA, Aerts HJW, Andrearczyk V, Apte A, et al. The image biomarker standardization initiative: Standardized quantitative radiomics for high-throughput image-based phenotyping. Radiology. 2020;295:328–338. PMID: 32154773

19. Zijdenbos AP, Dawant BM, Margolin RA, Andrew CP. Morphometric Analysis of White Matter Lesions in MR Images: Method and Validation. IEEE Trans Med Imaging. 1994;13:716–724. PMID: 18218550

20. Liljequist D, Elfving B, Roaldsen KS. Intraclass correlation – A discussion and demonstration of basic features. PLoS ONE. 2019;14:e0219854. PMID: 31329615

21. Koo TK, Li MY. A Guideline of Selecting and Reporting Intraclass Correlation Coefficients for Reliability Research. J Chiropr Med. 2016;15:155–163. PMID: 27330520

22. Bill McNeese (2018) Are the Skewness and Kurtosis Useful Statistics? via https://www.spcforexcel.com/knowledge/basic-statistics/are-skewness-and-kurtosis-useful-statistics#kurtosis Accessed 7 May 2022

23. Davnall F, Yip CSP, Ljungqvist G, Selmi M, Ng Francesca, Sanghera B, et al. Assessment of tumor heterogeneity: An emerging imaging tool for clinical practice? Insights Imaging.2012;3:573–589. PMID: 23093486

24. Shaghaghi M, Aliyari Ghasabeh M, Ameli S, Ghadimi M, Hazhirkarzar B, Habibabadi RR, et al. Post-TACE changes in ADC histogram predict overall and transplant-free survival in patients with well-defined HCC: a retrospective cohort with up to 10 years follow-up. Eur Radiol. 2021;31:1378–1390. PMID: 32894356

25. B Palcic, L D Skarsgard. Reduced oxygen enhancement ratio at low doses of ionizing radiation. Radiat Res. 1984;100:328–39. PMID: 6494444

26. Copyright 2016, pyradiomics community via <https://pyradiomics.readthedocs.io/en/latest/features.html> Accessed 7 May 2022

27. Pathak R, Ragheb H, Thacker NA, Morris DM, Amiri H, Kuijer J, et al. A data-driven statistical model that estimates measurement uncertainty improves interpretation of ADC reproducibility: A multi-site study of liver metastases. Sci Rep. 2017;7:1–10. PMID: 29075009

28. Papadopoulou I, Stewart V, Barwick TD, Edward Park WH, Soneji N, Rockall AG, et al. Post – Radiation Therapy Imaging Appearances in Cervical Carcinoma. RadioGraphics. 2016;36:538–553. PMID: 26849153

29. Addley HC, Vargas HA, Moyle PL, R Crawford, E Sala, et al. Pelvic imaging following chemotherapy and radiation therapy for Gynecologic malignancies. Radiographics. 2010;30:1843–1856. PMID: 21057123

30. Meng J, Liu S, Zhu L, Zhu L, Wang H, Xie L, et al. Texture Analysis as Imaging Biomarker for recurrence in advanced cervical cancer treated with CCRT. Sci Rep. 2018;8:1–9. PMID: 30061666

31) Meng J, Zhu L, Zhu L, Xie L, Wang H, Liu S, et al. Whole-lesion ADC histogram and texture analysis in predicting recurrence of cervical cancer treated with CCRT. Oncotarget. 2017;8:92442–92453. PMID: 29190929

32. Xue H, Ren C, Yang J, Sun Z, Li S, Jin Z, et al. Histogram analysis of apparent diffusion coefficient for the assessment of local aggressiveness of cervical cancer. Arch Gynecol Obstet. 2014;290:341–348. PMID: 24687746

33. Makino H, Kato H, Furui T, Morishige K, Kanematsu M. Predictive value of diffusion-weighted magnetic resonance imaging during chemoradiotherapy for uterine cervical cancer. J Obstet Gynaecol Res. 2014;40:1098–1104. PMID: 24320754

**Supporting information**

**S1 Table. Clinical data and the results of univariate analyses of SqCC patients only.**

**S2 Table. The image parameters with high AUCs in the 49 SqCC patients.**

**S1 Fig. Kaplan-Meier plots for SqCC patients.**

Log-rank *p* values were calculated.

*Abbreviations:* glcm = Grey level co-occurrence matrix

**S3 Table. The AUCs of the all image parameters.**
