# Supplementary material for: Prognosis prediction of uterine cervical cancer using changes in the histogram and texture features of apparent diffusion coefficient during definitive chemoradiotherapy
Source: PLoS One. 2023 Mar 31;18(3):e0282710. doi: 10.1371/journal.pone.0282710 (PMC10065283; doi:10.1371/journal.pone.0282710)
Supplement: Attachment — Submitted filename: Response to reviewers_plos20221029.docx [file pone.0282710.s008.docx]

Dr. Emily Chenette, Editor-in-Chief

Dr. Ngie Min UngAcademic Editor

PLOS ONE

Dear Editor and Reviewers:

Please find enclosed our revised manuscript entitled “**Prognosis prediction of uterine cervical cancer using changes in the histogram and texture features of apparent diffusion coefficient during definitive chemoradiotherapy**”; Ref. No.: PONE-D-22-13961

We thank the Editor-in-Chief and the reviewers for their carefully reading our manuscript and insightful comments. We have addressed the comments and revised the manuscript accordingly. Also, our point-by-point responses to the reviewers’ comments below have been included.

We trust that the revised version is now suitable for publication.

Sincerely yours,

**Point-by-point Responses to the Reviewers**

We express our appreciation to the reviewers for his/her insightful comments, which have helped us significantly improve our paper. Below, please find a point-by-point response to the comments, with the comments in italic font and our responses in normal font. Revised areas in the manuscript are indicated in red font.

**Response to Reviewer #1:**

*Why are tumor markers listed ? Are they specific of SCC Cervix cancer ?*

Response:

Tumor markers (CEA, CA19-9, CA125, SCC antigen) were acquired every 2–3 months during follow-up in order to detect recurrence as early as possible. In particular, SCC antigen (for Squamous cell carcinoma) and CA125 (for adenocarcinoma) are helpful for detecting recurrence with no symptoms [1,2].

*Why was CT done - to look for nodes ? If yes what was the criteria ? Why and how was PET-CT used ? Please specify ? Was it still biopsied after PET- What was SUV cut off ? What was the standard of truth - HPE or Clinical assessment ?*

Response:

CT and PET-CT were taken to look for lymph node metastasis and distant metastasis [3]. PET-CT can be added when other imaging findings are equivocal for recurrence [4]. We did not use specific SUV cut-off values. SUV value was one of the imaging factors used to determine the recurrence and disease extent. Recurrences were comprehensively diagnosed based on physical examination, imaging, and clinical course after discussion among gynecologists, radiologists, and pathologists.

I have added a statement about how to diagnose recurrence.

*This has to be one of the major flaws in this manuscript which wasn't addressed . What was the significance of a "2 year recurrence " follow up ? This has to be elaborated more in all parts of the manuscript !*

Response:

A systematic review showed that 62%–89% of cases of cervical cancer recurrence were detected within 2 years of primary treatment [5]. According to these data, ESMO (European Society for Medical Oncology) guidelines recommend follow-up visits every 3–6 months in the first two years and every 6–12 months in years 3–5 [3]. We decided our outcome as recurrence at 2 years based on these data. We have added the statement to our manuscript. Two-year recurrence-free survival is a common index for assessing the prognosis of uterine cervical cancer, and many previous studies used it [6,7].

*Another major flaw - Mentioned in manuscript that areas of necrosis were omitted at time of segmentation- Past literature shows necrosis was infact more inclusive of overall prognosis .*

Response:

We excluded necrotic and cystic areas because they increase outliers, especially during radiotherapy. Many previous studies dealing with MRI of cervical cancer use VOIs without necrosis [6,8-10].

**Response to Reviewer #2:**

*1) The English language could be proofread - This does not affect the clarity of contents but could nevertheless be improved.*

*a. There are a few instances where the spelling is following both the American and British styles (tumor vs tumour, gynecology vs gynaecology, hemorrhage vs haemorrhage).*

Response:

We have rewritten the manuscript in American style.

*b. There are few sentences started with abbreviations, in which this normally should be avoided in a formal and scientific writing. I am giving two examples from the manuscript;1) DSCs of the 1st and 2nd MRI were good (0.852 and 0.763), while DSCs of the 3rd and 4th MRI were poor... 2) Glcm_ClusterShade of 2nd and 3rd MRIs and the glcm_ClusterShade change 2nd –3rd had poor reliability...*

Response:

We have rewritten the sentences starting with abbreviations.

*2) I find the writing of references does not follow the correct format. I suggest the author to review and check the correct format for reference writing according to journal requirements. In addition to this, each reference in the sentences does not link to the reference list. This arises difficulties to the reviewer to cross-check the reference. It would be good if this was included in the manuscript. Author can use the References Tab in the words document for cross-reference link.*

Response:

We have reconfirmed the format of the references and corrected them.

We have also added links for each reference, and trust that these will make it easier for the reviewers to cross-check the references.

*3) This manuscript needs to be re-written in more rigorous and scientific manner. Although the study provides meaningful data and information, I believe the clarification on data presented in this manuscript is being blinded in the use of non-scientific manner writing.*

Response:

Thank you for your advice. We have rewritten or added statements according to your comments.

*Specific Comments: Please refer to the comment boxes inserted in the uploaded reviewer's attachment.*

Response:

We have rewritten or added statements to the manuscript and replied to each comment box.

1.	Gadducci A, Tana R, Cosio S, Genazzani AR. The serum assay of tumour markers in the prognostic evaluation, treatment monitoring and follow-up of patients with cervical cancer: a review of the literature. Crit Rev Oncol Hematol. 2008;66(1):10-20.

2.	Fu J, Wang W, Wang Y, Liu C, Wang P. The role of squamous cell carcinoma antigen (SCC Ag) in outcome prediction after concurrent chemoradiotherapy and treatment decisions for patients with cervical cancer. Radiat Oncol. 2019;14(1):146.

3.	Marth C, Landoni F, Mahner S, McCormack M, Gonzalez-Martin A, Colombo N, et al. Cervical cancer: ESMO Clinical Practice Guidelines for diagnosis, treatment and follow-up. Ann Oncol. 2017;28(suppl_4):iv72-iv83.

4.	Cibula D, Pötter R, Planchamp F, Avall-Lundqvist E, Fischerova D, Haie Meder C, et al. The European Society of Gynaecological Oncology/European Society for Radiotherapy and Oncology/European Society of Pathology guidelines for the management of patients with cervical cancer. Radiotherapy and Oncology. 2018;127(3):404-16.

5.	Elit L, Kennedy EB, Fyles A, Metser U. Follow-up for cervical cancer: a Program in Evidence-Based Care systematic review and clinical practice guideline update. Curr Oncol. 2016;23(2):109-18.

6.	Zhao B, Cao K, Li XT, Zhu HT, Sun YS. Whole lesion histogram analysis of apparent diffusion coefficients on MRI predicts disease-free survival in locally advanced squamous cell cervical cancer after radical chemo-radiotherapy. BMC Cancer. 2019;19(1):1-7.

7.	Lin G, Yang LY, Lin YC, Huang YT, Liu FY, Wang CC, et al. Prognostic model based on magnetic resonance imaging, whole-tumour apparent diffusion coefficient values and HPV genotyping for stage IB-IV cervical cancer patients following chemoradiotherapy. European Radiology. 2019;29(2):556-65.

8.	Bae JM, Kim CK, Park JJ, Park BK. Can diffusion-weighted magnetic resonance imaging predict tumor recurrence of uterine cervical cancer after concurrent chemoradiotherapy ? Abdominal Radiology. 2016;41(8):1604-10.

9.	Gu Kw, Kim CK, Choi CH, Yoon YC, Park W. Prognostic value of ADC quantification for clinical outcome in uterine cervical cancer treated with concurrent chemoradiotherapy. European Radiology. 2019;29(11):6236-44.

10.	Lee J, Kim CK, Park SY. Histogram analysis of apparent diffusion coefficients for predicting pelvic lymph node metastasis in patients with uterine cervical cancer. Magnetic Resonance Materials in Physics, Biology and Medicine. 2020;33(2):283-92.
